# Evaluation of the *rbcL* marker for metabarcoding of marine diatoms and inference of population structure of selected genera

**DOI:** 10.3389/fmicb.2023.1071379

**Published:** 2023-03-02

**Authors:** Timotej Turk Dermastia, Ivano Vascotto, Janja Francé, David Stanković, Patricija Mozetič

**Affiliations:** ^1^Marine Biology Station Piran, National Institute of Biology, Piran, Slovenia; ^2^Jožef Stefan International Postgraduate School, Ljubljana, Slovenia; ^3^Department of Organisms and Ecosystems Research, National Institute of Biology, Ljubljana, Slovenia

**Keywords:** *rbcL*, metabarcoding, monitoring, diatoms, population genetics, *Pseudo-nitzschia*, Adriatic

## Abstract

Diatoms are one of the most important phytoplankton groups in the world’s oceans. There are responsible for up to 40% of the photosynthetic activity in the Ocean, and they play an important role in the silicon and carbon cycles by decoupling carbon from atmospheric interactions through sinking and export. These processes are strongly influenced by the taxonomic composition of diatom assemblages. Traditionally, these have been assessed using microscopy, which in some cases is not reliable or reproducible. Next-generation sequencing enabled us to study diversity in a high-throughput manner and uncover new distribution patterns and diversity. However, phylogenetic markers used for this purpose, such as various 18S rDNA regions, are often insufficient because they cannot distinguish between some taxa. In this work, we demonstrate the performance of the chloroplast-encoded *rbcL* marker for metabarcoding marine diatoms compared to microscopy and 18S-V9 metabarcoding using a series of monthly samples from the Gulf of Trieste (GoT), northern Adriatic Sea. We demonstrate that *rbcL* is able to detect more taxa compared to 18S-V9 metabarcoding or microscopy, while the overall structure of the diatom assemblage was comparable to the other two methods with some variations, that were taxon dependent. In total, 6 new genera and 22 new diatom species for the study region were identified. We were able to spot misidentification of genera obtained with microscopy such as *Pseudo-nitzschia galaxiae*, which was mistaken for *Cylindrotheca closterium*, as well as genera that were completely overlooked, such as *Minidiscus* and several genera from the Cymatosiraceae family. Furthermore, on the example of two well-studied genera in the region, namely *Chaetoceros* and particularly *Pseudo-nitzschia*, we show how the *rbcL* method can be used to infer even deeper phylogenetic and ecologically significant differences at the species population level. Despite a very thorough community analysis obtained by *rbcL* the incompleteness of reference databases was still evident, and we shed light on possible improvements. Our work has further implications for studies dealing with taxa distribution and population structure, as well as carbon and silica flux models and networks.

## 1. Introduction

Diatoms belong to the phylum Ochrophyta and are an obligate autotrophic group. According to some estimates, they are responsible for up to 40% of the primary productivity of the ocean (Nelson et al., 1995). They are a highly ecologically successful and diverse group that also plays a key role in the biogenic silica cycle by forming silicate frustules that protect their cells. They also act as ballast, contributing significantly to carbon export and the biological carbon pump, especially in the oligotrophic stratified ocean (Nelson et al., 1995). This is also directly influenced by their diversity, as diatoms vary in the size, shape, and thickness of their frustules (Tréguer et al., 2018 and references therein). These frustules are also the key morphological features for species identification. The occupation of different ecological niches, from oceanic, coastal, benthic and epiphytic environments, has led to a wide distribution of this group (Round et al., 1990). However, the actual number of species is very difficult to estimate because cultivation and detailed morphological or genetic analysis are often required to identify species. New groups and species are constantly being discovered through the elucidation of phylogenetic relationships among different groups, while high-throughput genetic techniques and advanced imaging techniques that help decipher differences among species and reveal diversity have only recently begun to be applied on a large scale. Phytoplankton monitoring, which helps to detect changes in phytoplankton communities and establish long-term ecological changes and processes, utilizes so-called long-term ecological research (LTER) sites (Edwards et al., 2010) where a wide range of data, including phytoplankton community structure, is collected over long periods of time. The role of such sites has been shown to be immensely important, although they are often underfunded and/or neglected as “routine monitoring” (Zingone et al., 2019). Phytoplankton monitoring has traditionally relied on phytoplankton counts, largely based on the Utermöhl method (Utermöhl, 1958). However, in recent years, many LTER sites, including those in the Mediterranean, have incorporated different molecular methods into their programs, the most powerful of which is environmental DNA (eDNA) analysis using metabarcoding (Piredda et al., 2017; Stern et al., 2018; Armeli Minicante et al., 2020). Metabarcoding has gained appeal as it offers several advantages for natural community analysis compared to traditional methods (De Vargas et al., 2015; Malviya et al., 2016; Penna et al., 2017; Piredda et al., 2018; Cristescu, 2019). These include high throughput; scalability, i.e., large-scale sampling campaigns and analyzes are possible and comparable to small-scale campaigns; interoperability, i.e., datasets can be compiled based on the same bioinformatics pipelines and are thus comparable, while data obtained through counts or observations are always observer-dependent; detection of rare and cryptic taxa; detection of non-indigenous species. On the other hand, eDNA analysis brings its own problems. First, it depends on reference databases, which are usually not complete, since most marine organisms have not yet been cultured (Weigand et al., 2019). However, even those that have been cultured and barcoded may have considerable genetic diversity, leading to conceptual and technical problems in assigning species or even higher taxonomic levels. Most studies focus on the V4 or V9 regions of the universal 18S eukaryotic domain because it allows comparison across different taxonomic levels and eukaryotic groups (De Vargas et al., 2015; Piredda et al., 2017). However, 18S is not a very informative marker and species-level resolution in case of diatoms is often difficult to achieve even when the entire gene is considered (Moniz and Kaczmarska, 2009; Guo et al., 2015). In addition, the definition of molecular species is problematic since usually no ecological and morphological reference can be defined, especially with environmental samples. To overcome this problem, the operational taxonomic unit (OTU) concept was introduced (Caron et al., 2009), which enables clustering of similar sequences to retrieve relative species-like resolution, while accounting for sequencing errors. However, this method inevitably leads to loss of information obtained by sequencing, while it does not account for sequences that are potentially shared among different species that get clustered within the same OTU. To prevent information loss, error corrected sequences–using algorithms such as *dada2* (Callahan et al., 2016)–can be clustered at 100% identity to produce amplicon sequence variants (ASVs). Conversely, in order to increase the resolution for species detection, other more variable markers can be used, but so far we have seen limited application in the field of marine phytoplankton monitoring. For example, the *rbcL* marker has previously been used in metabarcoding of freshwater diatoms (Vasselon et al., 2017b) and in reconstructing clone libraries of chromophytic phytoplankton in mangrove forests (Samanta and Bhadury, 2014). The resolution of the 312 bp barcoding region (Rimet et al., 2016) has been demonstrated on the example of the marine diatom *Pseudo-nitzschia* (Turk Dermastia et al., 2020), while different regions of the marker have been evaluated several times for barcoding suitability of diatoms (Daugbjerg and Andersen, 1997; Watson and Tabita, 2006; Guo et al., 2015). They all showed that the marker is more suitable for barcoding compared to 18S, especially for smaller fragments. On the other hand, it may lack resolution compared to the more diverse COI and ITS-2 markers, although these two often exhibit difficulties in amplification and alignment (Moniz and Kaczmarska, 2009). Based on these findings, *rbcL* appears to be an ideal candidate for marine diatom metabarcoding.

In the Gulf of Trieste, the northernmost part of the Adriatic Sea, phytoplankton have been consistently monitored using light microscopy for almost 40 years (Cabrini et al., 2012; Mozetič et al., 2012; Cerino et al., 2019). This long period is also characterized by a significant decline in phytoplankton biomass observed throughout the northern Adriatic basin over the last two decades (Mozetič et al., 2012; Brush et al., 2021), largely due to phosphorus limitation exacerbated during the drought in major rivers (Mozetič et al., 2010; Brush et al., 2021). The observed regime shift is clearly reflected in the changes in the main phytoplankton groups and in species diversity, including diatoms, which account for the largest proportion of the total biomass (Vascotto et al., 2021). Thus, the typical pattern of diatom assemblage in recent times is characterized by two seasonal peaks in summer and fall (Brush et al., 2021). The typical spring bloom (February–March), which was predominant in earlier decades (Cabrini et al., 2012), decreased and developed in later months (May–June), but with lower abundances and different species. For example, the diatom *Skeletonema marinoi* bloomed in late winter and early spring until 2000 and was in later years replaced by blooms of smaller diatoms of the genus *Chaetoceros*, which may be more efficient in an oligotrophic environment (Cabrini et al., 2012). In addition, diversity can also be disrupted by the introduction of non-indigenous species (NIS), which in some cases can even be harmful. One such NIS, *Pseudo-nitzschia multistriata*, was recently found in Adriatic ports (Mozetič et al., 2019). To follow such changes in more detail, a deeper, taxonomist-independent, high-throughput analysis of the phytoplankton community at this stage is highly desired and necessary.

In this work, we evaluated the *rbcL* plastid marker to describe diatom assemblages in a half year-long study and compared it to 18S-V9 metabarcoding and light microscopy. The study is the first of its kind in the area and demonstrates the power of molecular monitoring strategies as previously unknown community members were discovered. We have also evaluated the *rbcL* marker as a tool for analyzing population structure by analyzing haplotype composition in diatoms of the genera *Pseudo-nitzschia* and *Chaetoceros* and comparing the resulting composition to known haplotype structure (Turk Dermastia et al., 2020).

## 2. Methods

### 2.1. Sampling campaign

**Figure 1:**
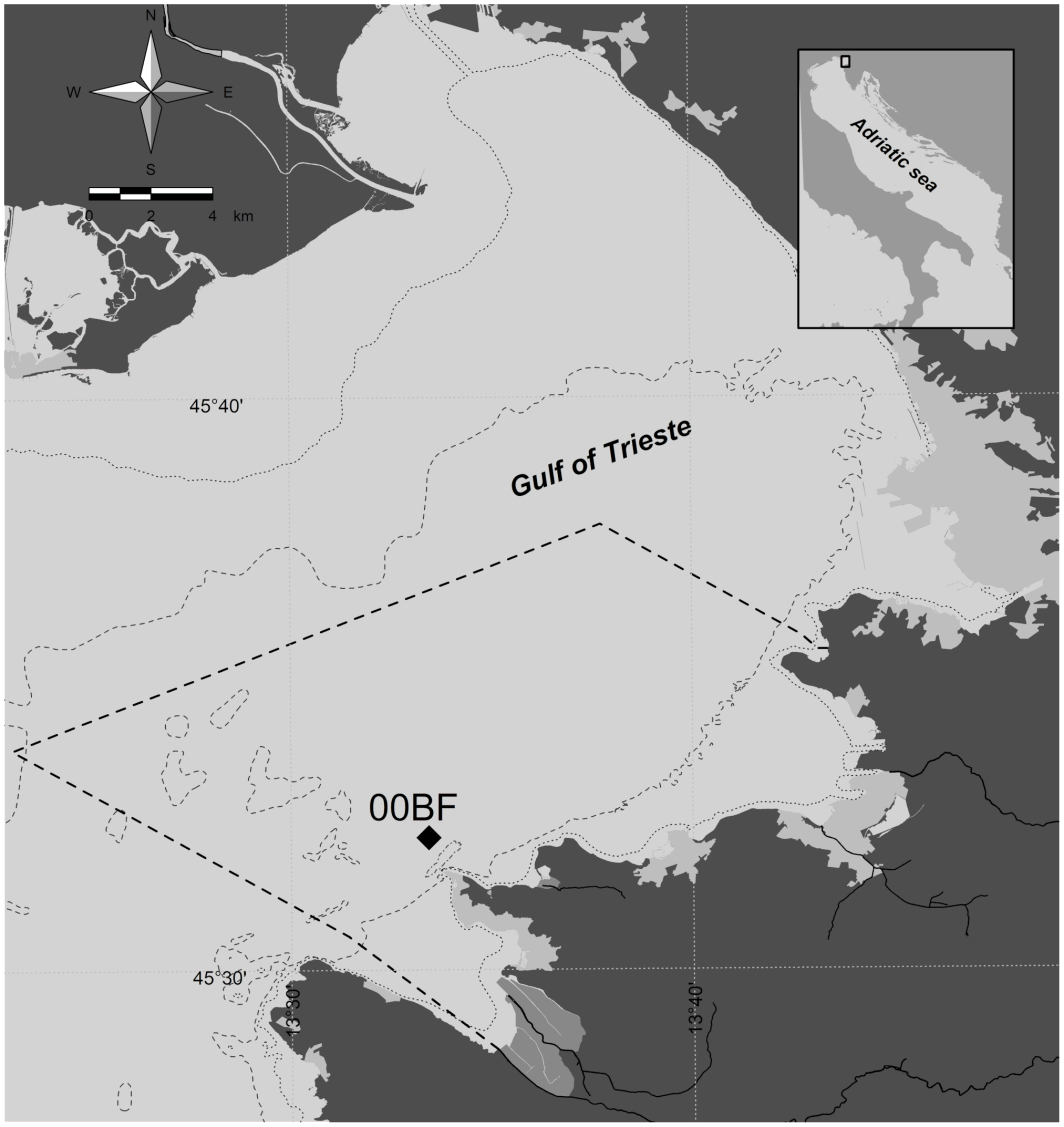
Sampling location. Dashed line represents the border of the Slovenian territorial sea.

Samples were collected from September 2019 to February 2020 and an additional one in October 2020 (October-20) at the 00BF station (LTER-SI; Figure 1). A total of 36 samples were collected. Seawater was collected at 0 m and 5 m using 6-liter Niskin bottles. For metabarcoding, 1 l of seawater from both depths was filtered in triplicate on 0.8 μm polycarbonate filters without prefiltration. The filters were frozen and stored at −80°C. Simultaneous phytoplankton counts using the Utermöhl technique (Utermöhl, 1958) were performed as part of ongoing monitoring activities.

Sampling in October-20 was conducted using a phytoplankton net by towing the net horizontally five times from the depth of 5 m. The collected sample was subsequently filtered the same way as described above.

Alongside sampling, several environmental parameters were measured. CTD temperature and salinity profiles were obtained with a SBE 19plus SEACAT multiparametric probe. Discrete seawater samples were collected with 6-liter Niskin bottles at two depths (0.5, 5). Dissolved inorganic nutrient concentrations were determined colorimetrically on filtered samples with a QuAAtro (Seal Analytical), according to Hansen and Koroleff (1999).

### 2.2. Extraction of DNA for metabarcoding analysis

DNA from environmental samples destined for metabarcoding was extracted in two ways, as samples were filtered in triplicate. After a filter crushing step, in which the tubes containing the filters were immersed in liquid nitrogen and crushed into small pieces using a sterile metal spatula, one replicate was extracted using the E.Z.N.A Mollusc DNA Kit according to the manufacturer’s guidelines. The other two were extracted using the phenol-chloroform extraction procedure described in the protocol of Angel (2012) and modified for extraction from filters. These were immersed in liquid nitrogen and crushed with a sterile metal spatula. 1 ml of 120 mM phosphate buffer (pH 8) and 125 μl of TNS buffer (500 mM Tris base, 100 mM NaCl, 10% SDS) were added to the crushed filters. Extraction then proceeded as in the original procedure. DNA concentration and quality of both phenol and E.Z.N.A replicates were measured by Nanodrop spectrophotometer and on an agarose gel (1%) by electrophoresis (60 V, 1 h). The reason for different extraction approaches was to achieve maximal DNA recovery and amplification success, and not for comparative purposes.

### 2.3. Amplification and Illumina MiSeq sequencing

Two markers were chosen for metabarcoding, namely the 150 base pair (bp) 18S-V9 region and a ~312 bp barcode within the *rbcL* chloroplast gene. The primers used to amplify the 18S gene were the universal eukaryotic 18S-V9F (TTGTACACACCGCCCGTCGC) and 18S-V9R (CCTTCYGCAGGTTCACCTAC; Piredda et al., 2017). Libraries for the *rbcL* barcode were built using the diatomspecific primers 708F-DEG (AGGTGAAGYWAAAGGTTCWTAYTTAAA) and R3-DEG (CCTTCTAATTTACCWACWACWG), both modified from Vasselon et al. (2017a). All primers were modified according to the Illumina protocol by adding universal Illumina tails. The initial amplification step using primers with Illumina adapters was performed by the authors. Samples that failed to amplify were not further considered, while those with visible amplification products were sent to BMR Genomics srl (Padua, Italy) where further library preparation and sequencing were performed. Sequencing libraries for the entire series of samples were generated with 18S, whereas we were unable to obtain sequences from October with *rbcL*. The list of samples and associated metadata can be found in Supplementary Table S1. 18S-V9 amplicons were sequenced with 2 x 150 bp MiSeq reagent kits, while *rbcL* amplicons were sequenced using 2 x 300 bp kits at 300 and 600 cycles, respectively. Both runs produced roughly 1.5 M reads, but the 18S run was sequenced together with samples not reported in this study which resulted in a lower average number of reads per sample.

### 2.4. Bioinformatics analysis

The sequences provided by BMR Genomics had already been demultiplexed with primers removed. For inference of diatom assemblage data, sequence denoising and taxonomy assignment were performed using the *dada2* software package (Callahan et al., 2016) embedded in the R software framework (R Core Team, 2019). Reads were filtered and pruned based on quality scores. The quality of reads was good, except at the beginning of the reads. Error rates were assumed using the implemented *dada2* algorithm and applied to the sample inference step where ASVs and their relative abundance are derived. For both markers, 13 bases of the 5′ ends were trimmed while the length was eventually truncated to 150 bp with the first run and to 300 bp with the second run for *rbcL*. The maxEE score was derived with Figaro software (Weinstein et al., 2019) and set to 2 for both reverse (RR) and forward reads (FR). Reads with ambiguous nucleotides were excluded. FR and RR were then merged. The sequence table obtained was cleaned of chimeric sequences and singletons. To further decrease noise we applied the LULU curation algorithm (Frøslev et al., 2017) for inferring erroneous ASVs based on co-occurrence and similarity. The minimum threshold of sequence similarity for considering any ASV as an error of another was set to 97% but the result was the same even with default settings (84%). All other parameters were left as default. Since *rbcL* is a coding gene, ASVs were manually analyzed for the presence of stop codons in translation. Sequences containing stop codons were removed. Codon entropy ratios between the entropy of position 2 and position 3 were calculated according to Turon et al. (2020) to determine the influence of the curation process. Sequences from two different depths were for the purpose of this study pooled by averaging to represent the monthly surface diatom assemblages, except in certain cases where fine-scale resolution was of interest.

For taxonomy assignment, two approaches were followed, both dealing with ASVs. The first was the naïve Bayesian classifier (NBC) with 50% bootstrap thresholds (NBC50) for classifying any given taxonomy rank implemented in the *dada2* package. 80% thresholds were also tested (NBC80) but are not reported. For the 18S marker, we used the PR2 database, version 4.12 (Guillou et al., 2013), while for *rbcL* we used the Rsyst::diatom database, version 9 (Rimet et al., 2016), which we modified by adding local taxa sequences obtained during this and previous works (Turk Dermastia et al., 2020, 2022), as well as other underrepresented marine diatom taxa, increasing the number of diatom taxa in the database to 1,454. The database used can be accessed through Zenodo (https://doi.org/10.5281/zenodo.7064747). An alternative taxonomy was assigned using local BLAST implemented in the software MALT (Herbig et al., 2016) with a cutoff value of 97% and a maximum E value of -40E10 and by saving the top 10 hits, using the top hit for visualization. Reference databases for 18S and *rbcL* BLAST were obtained from GenBank using the search terms available in the supplemental data (Code Piece 1). The resulting assignments were visualized in MEGAN (Huson et al., 2007, RRID:SCR_011942). 18S data was first cleared of metazoan sequences by applying filtration methods implemented in the R package *phyloseq* (McMurdie and Holmes, 2013). This data was used to visualize the protist assemblages, before proceeding with the analysis of diatoms. Diatom species accumulation curves were obtained with the *vegan* package (Oksanen et al., 2019) with the *rarecurve* function on species-agglomerated data, since ASVs are not directly comparable between different phylogenetic markers. Visualization, filtration and agglomeration of abundance data was performed using *phyloseq*, together with *ggplot2* (Wickham, 2016).

To infer the haplotype network of *Pseudo-nitzschia* we selected ASVs assigned to the *Pseudo-nitzschia* genus with BLAST. Haplotype networks were constructed using the *pegas* package in R (Paradis, 2018), and phylogenetic trees and heat maps were constructed and drawn using the *ape* (Paradis and Schliep, 2019, RRID:SCR_017343) and *ggtree* (Yu et al., 2016, RRID:SCR_018560) packages.

### 2.5. Comparative analysis of diversity

Since the 18S marker recovered the entire eukaryote community, we have for the purpose of this study filtered out all metazoan taxa at the first stage. These data from 18S served as an overview of the structure of the phytoplankton community and as a comparison to microscopy-derived structure. Two data normalization approaches were conducted. The first was the *χ*^2^ transformation implemented in the *decostand* function in *vegan*. The second, followed the normalization procedures described in Gloor et al. (2017) for treating high-throughput sequencing data as compositional based on the centered log-ratio (CLR) transformation (Aitchison, 1982). Here zeros were replaced by pseudocounts prior to the transformation but were then back-traced and replaced by zeros again.

The progression of diatom abundance was inspected based on the CLR values of the complete ASV datasets. α-diversity estimates including confidence intervals were calculated following the Aitchison log-ratio model for compositional data (Aitchison, 1982) using the *divnet* (Willis and Martin, 2022) and *breakaway* packages (Willis and Bunge, 2015). Richness, Shannon Diversity and Simpson’s Diversity were estimated for a series of methods and taxonomical approach pairs. The differences between different methods and approaches were tested using the TukeyHSD test. Based on these and previous results we selected methods and taxonomical approaches that were the most robust for each barcoding marker. These were then used in a β-diversity analysis and similarity assessment. For this process we used the R package *CoDaSeq* (Gloor et al., 2016) to perform the CLR transformation, followed by the calculation of expected CLR values with the *ALDEx2* package (Fernandes et al., 2014). A principal component analysis (PCA) was conducted on these data, where environmental variables were also fitted to the ordination using the *envfit* function implemented in *vegan* (Oksanen et al., 2019). Significance of differences was tested using the *anosim* function in the *vegan* package. Although the CLR transformation is stable when subsetting abundance data, we report the sequence of data filtering prior to transformation. First, data were agglomerated by depth, then diatom ASVs were selected and agglomerated to the genus level, prior to CLR transformation. Unassigned diatom ASVs were kept in the data. In addition, a correspondence analysis based on the weighted *χ*^2^ distances was also performed (Legendre and Legendre, 2012). Because the resulting ordination demonstrated a specific triangular shape, we used the *decorana* function to perform a detrended correspondence analysis as suggested by Legendre and Legendre (2012). Statistical significance of this relationship was tested using coinertia analysis implemented in the *ade4* package (Dray and Dufour, 2007) and Mantel test for matrix correlation. The comparative analysis was for the most part conducted using genus-agglomerated data, since many species could not be resolved using 18S-V9 and microscopy. The qualitative comparison of the relative abundance of genera was performed on non-normalized data.

## 3. Results

### 3.1. Diversity estimates from 18S and *rbcL* metabarcoding

The average number of reads per sample was about 50,000 ± 9,000 with 18S and 142,000 ± 50,000 with *rbcL* (Supplementary Table S1). 18S reads presented in this study amounted to roughly 600 k, while there were 1.5 M *rbcL* reads. Quality filtering, merging, denoising and chimera removal resulted in greatly reduced dataset of *rbcL* (20–30% retained) whereas with 18S the majority of reads were retained (80–90%). Diatom representation following NBC taxonomy implemented in *dada2* was low in 18S, as more than half of the ASVs belonged to metazoan organisms, while others belonged to other unicellular eukaryotes. Thus in some samples the number of diatom ASVs was less than 1%. With *rbcL*, for which primers targeting diatoms were used, the representation was much higher from 60 to 100%. With 18S irrespective of the taxonomy assignment method, 148 unique diatom ASVs were recovered. With *rbcL* this number depended on the assignment strategy. NBC50 assigned 1,113 ASVs while BLAST assigned 1,021. LULU curation was applied to both taxonomy datasets. Interestingly, the curation did not change the 18S data, whereas the *rbcL* data was greatly reduced following LULU, from the 1,113 ASVs assigned with NBC50 to 731 diatom ASVs. The result of LULU curation was further evaluated by calculating codon position entropies. The original dataset had a position2:position3 ratio of 1.09 suggesting a high error rate, since the second position is expected to be less variable. After LULU, this ratio dropped to 0.25. The species accumulation curves (Supplementary Figure S1) with 18S (NBC50) and *rbcL* LULU curated BLAST data show that with *rbcL* the sequencing depth was sufficient and saturation is achieved with a relatively low library size. On the other hand, with 18S the depth was at least for some samples not high enough. This was particularly evident with September samples. The number of species recovered was higher with *rbcL*.

**Figure 2:**
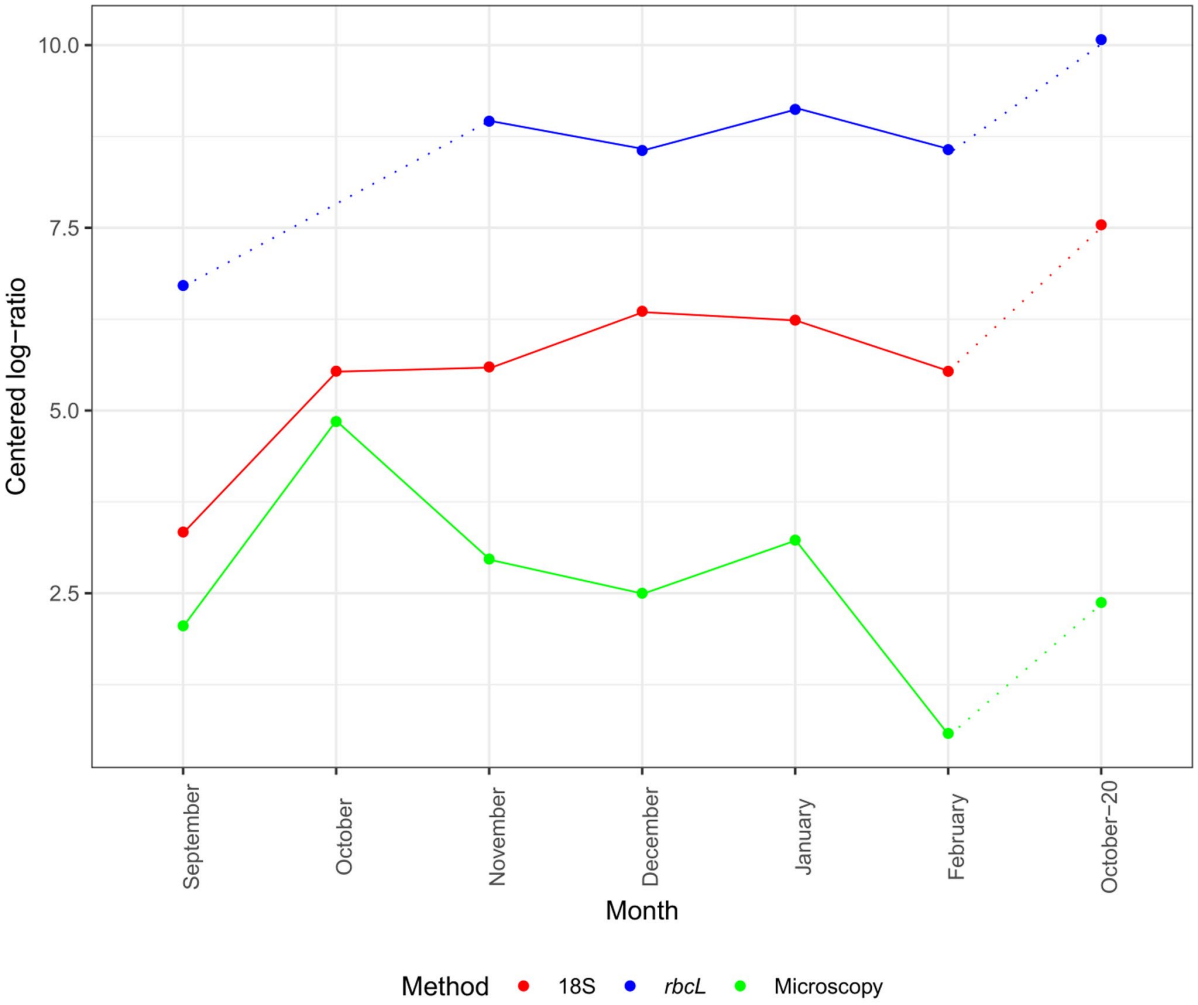
Monthly succession of diatom ASV number and cell abundance in the epipelagic layer (pooled depths) at certain months. Data are normalized with the CLR transformation. 18S-V9 data obtained with the NBC50 classifier, *rbcL* data curated with LULU and classified with BLAST. Dotted lines represent time point that are separated by more than 1 month.

Although our time series was not long, we can look at the changes in CLR-normalized diatom abundances and diatom classified amplicons by our methods (Figure 2). The inferred progression was similar between 18S and *rbcL* but diverged from that of microscopy. The absolute CLR values were the highest with *rbcL*. Both metabarcoding markers showed an increase in diatom classified amplicons from September to November, followed by a decline in December with *rbcL* and a continued increase with 18S. The momentary decline in December was registered also with microscopy. All three methods concurred in the decline of abundance in February. The higher abundance in October-20 was recorded with all three methods, as well but with the metabarcoding data the CLR value was the highest in the entire series. The peak of abundance with microscopy in October was not as profound with 18S, while we lack this data for *rbcL*.

**Figure 3:**
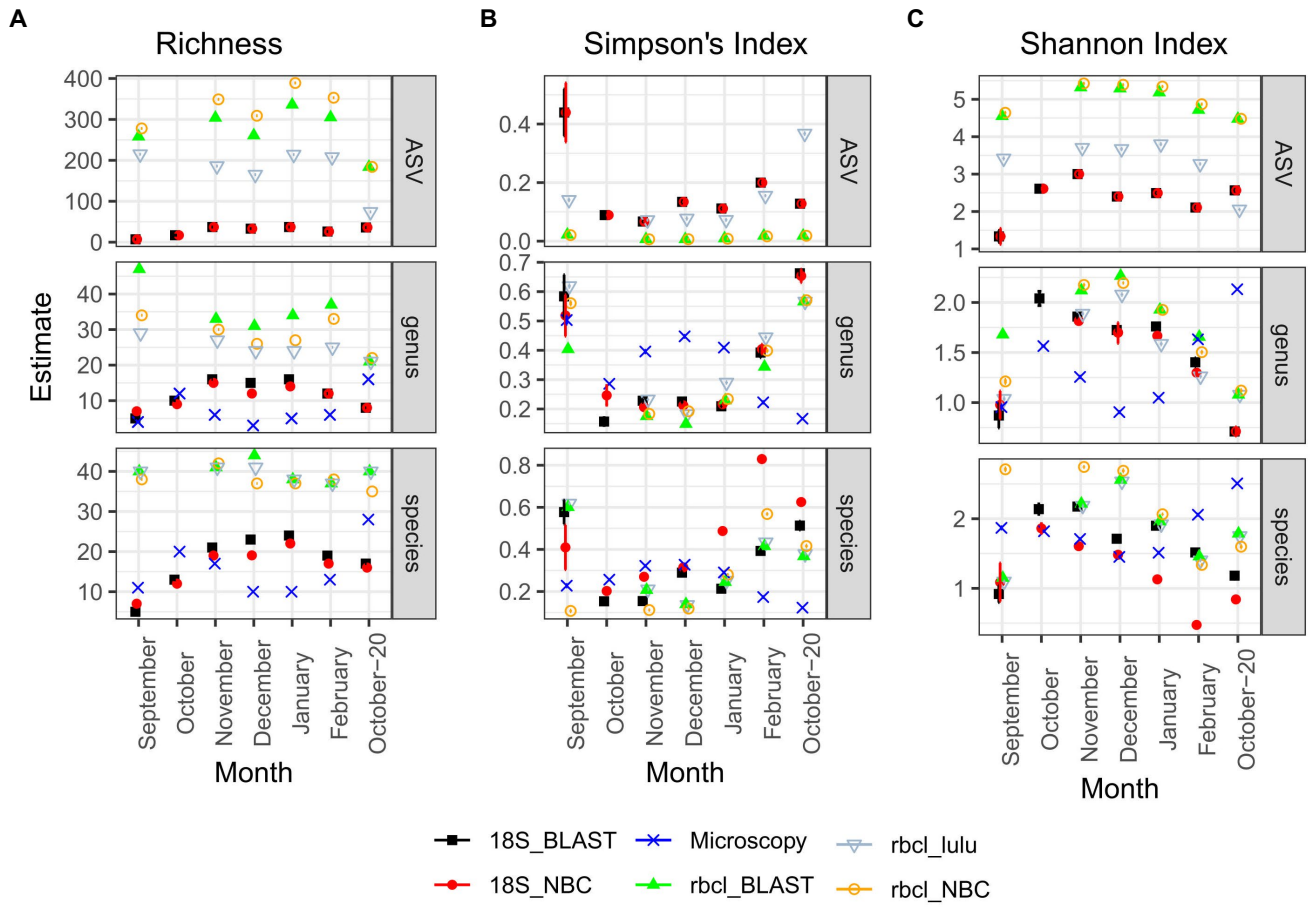
Diversity estimates obtained after CLR transformation with *divnet* and *breakaway* for different methods and taxonomy classification approaches. **(A)** Richness; **(B)** Simpson’s Index; **(C)** Shannon Index. Error bars are included but are visible only in a few cases. “Genus” and “species” panels represent data agglomerated to the genus or species levels, respectively. Microscopy data is by nature not included in the ASV column.

*rbcL* recovered the highest richness of ASVs, genera and species among all the methods (Figure 3A). Particularly on the ASV level, there were also significant differences between LULU curated and non-currated data and also between taxonomical approaches (Supplementary Figure S2). The differences between *rbcL* data and the other two methods were significant for all tested combinations. On the other hand, microscopy and 18S inferred richness were comparable and non-significantly different. The estimated Simpson’s Index (Figure 3B) was similar particularly between metabarcoding data, irrespective of the method, while the estimated index for microscopy data was different. Wherever the index for metabarcoding decreased it seemed to increase for microscopy. Therefore, with metabarcoding the least diverse samples were September and October-20, whereas with microscopy these were the winter months December and January. Nevertheless, these differences were not significant with the TukeyHSD test (Supplementary Figure S2), although the sample number was low. A similar case was observed for the Shannon Index (Figure 3C), although on the ASV level, the differences were significant between 18S and *rbcL* and also between LULU-currated and non-currated *rbcL* data. There were no significant differences on the genus level, while on the species level *rbcL* NBC data was different from 18S NBC. From these results, we established that LULU curated *rbcL* data were more robust and comparable to the data obtained by the other methods and were considered also in the β-diversity analyzes, while the others were not.

### 3.2. Diatom assemblage composition and comparison of methods

**Figure 4:**
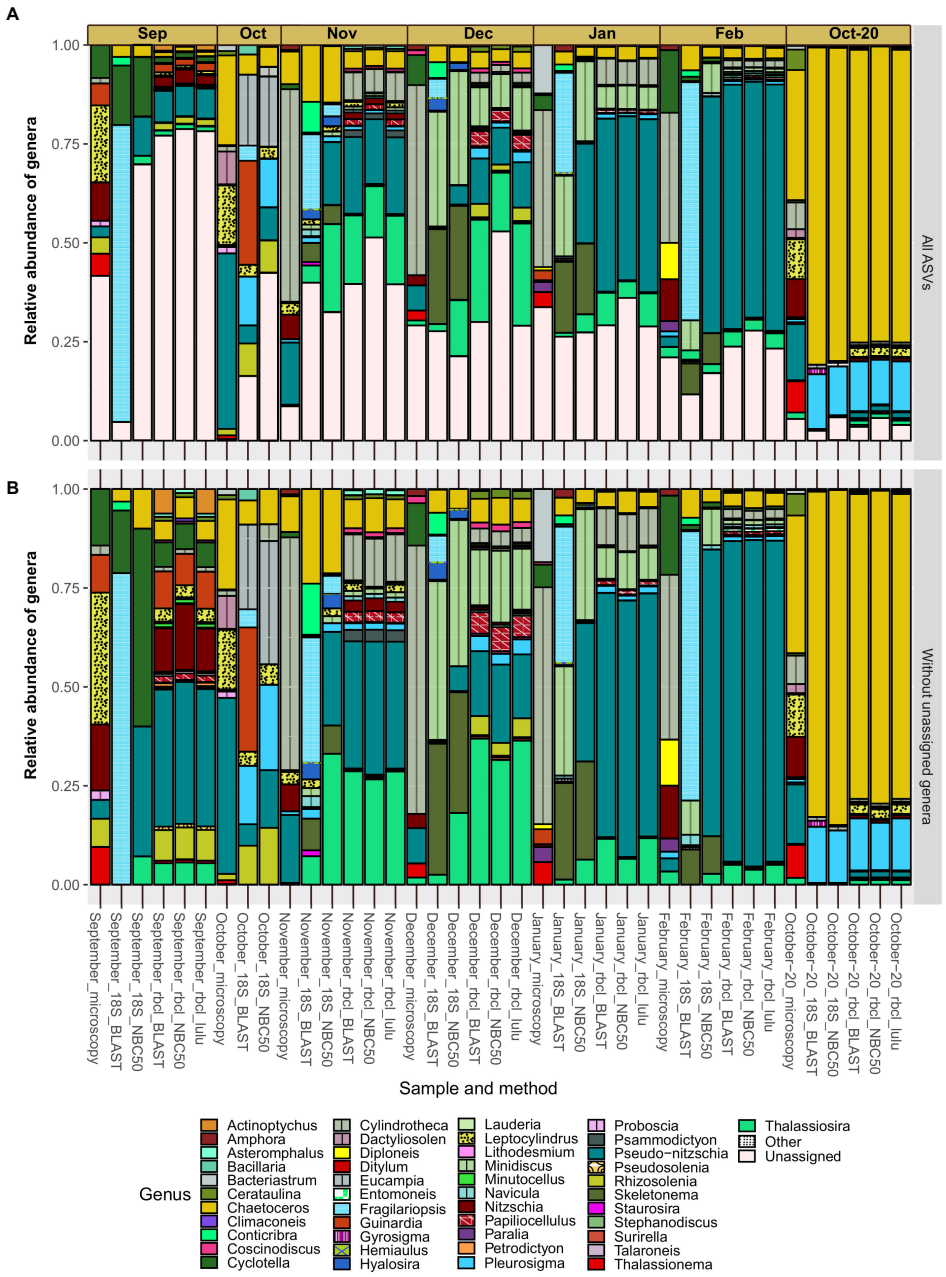
Monthly distribution and relative abundances of diatom genera recovered with microscopy, 18S-V9 and *rbcL* using different taxonomy classification approaches. Assemblages with included diatom ASVs with unassigned genera **(A)** and without unassigned genera **(B)**. Genera classified as “Other” had less than 50 reads in any sample and include: *Cocconeis*, *Cyclophora*, *Cymatosira*, *Extubocellulus*, *Gedaniella*, *Grammonema*, *Halamphora*, *Licmophora*, *Meuneria*, *Plagiotropis* and *Tryblionella*.

The relative abundance of genera differed on the qualitative scale quite substantially between the applied methods (Figure 4). The most obvious differences are between microscopy and each of the metabarcoding markers. The taxonomy of *rbcL* data did not differ largely between BLAST and NBC50 and interestingly, despite the clear change in diversity estimates not with LULU curated data. 18S data with BLAST taxonomy differed from 18S data with NBC50 taxonomy, particularly for a few genera. For example, ASVs classified as *Pseudo-nitzschia* with NBC50 were consistently classified as *Fragilariopsis* with BLAST. When we inspected these ASVs, we realized that they were also classified as *Pseudo-nitzschia* with identical BLAST scores. Given that *rbcL* data also recovered similar percentages of *Pseudo-nitzschia* in these months, we believe that NBC50 classification in the case of 18S was more appropriate. Similarly, BLAST did not identify *Cylindrotheca* as the first hit, but close examination showed that sequences classified as *Bacillaria* had the same BLAST scores and 100% identity with both *Cylindrotheca* and *Bacillaria*. A total of 18 and 26 genera were found using 18S NBC50 and 18S BLAST, respectively; 48, 43 and 42 with *rbcL* NBC50, BLAST and LULU, respectively; and 16 with microscopy. From this, it follows that 18S and microscopy were more comparable, but bear in mind, that sequencing was much deeper and targeting diatoms with *rbcL*.

The proportion of diatom ASVs without genus assignments (Figure 4A) was largely homogenous between the methods. This proportion was especially high in certain months such as September, where it reached almost 80%. Since the various methods, including microscopy, showed similar proportions of unassigned genera and because these proportions seemed unrelated to sequencing depth (e.g., the month of September), we hypothesize this is not a result of incomplete reference databases, but rather represent undescribed diatom diversity. The relative abundances of the most represented genera were comparable at least between *rbcL* and 18S (NBC50). These include *Chaetoceros*, *Minidiscus*, *Pseudo-nitzschia* and *Thalassiosira*. *Minidiscus* represented important relative abundances and is one of the genera that was never found in the studied region before. These are one of the smallest diatoms and are difficult to identify with light microscopy. Particularly high relative abundances of this genus were recorded in December and January. One clear difference between the methods is the large proportion of *Skeletonema* assigned reads with 18S data, but a complete lack of this genus in *rbcL* as well as in microscopy data. Another distinctive feature between the microscopic data and metabarcoding data is the seeming substitution of *Pseudo-nitzschia* in metabarcodes with *Cylindrotheca* in microscopy. This is perhaps not surprising as the majority of *Pseudo-nitzschia* ASVs belonged to *P. galaxie*, which could have been easily misidentified for *C. closterium* in preserved microscopy samples.

September showed a very similar assemblage recovered by microscopy and *rbcL*, where relative abundances of certain genera were also in accordance (*Cyclotella*, *Guinardia*, *Cylindrotheca*, *Nitzschia*, *Rhizosolenia*). Here, 18S underperformed but this was likely also to low diatom read recovery in general. The October samples where we lacked *rbcL* data, showed some similarity between microscopy and 18S but also some notable differences, for example the relatively large proportion of *Guinardia* ASVs recovered with 18S-BLAST. Fall samples showed high diversity with a more even distribution of abundance. In November and December, there was an increase in smaller taxa such as *Thalassiosira* and *Minidiscus* compared to the month of October. *Pseudo-nitzschia* also occupied a larger proportion in the fall, gradually increasing during the winter months. In February, the relative abundance of this genus exceeded 50% of the diatom assemblage, although this still represented only about 10% of the total phytoplankton community (Supplementary Figure S3). *Skeletonema* also occupied significant relative abundances in winter months, but only with 18S data. The most uniform month considering metabarcoding was October-20, for which the samples were obtained differently than in the other months. Here there was a prevalence of *Chaetoceros* and *Pleurosigma* ASVs. *rbcL* also recovered ASVs that belonged to genera also detected by microscopy, namely *Thalassiosira*, *Nitzschia*, *Pseudo-nitzschia* and *Leptocylindrus*. *Bacteriastrum* was detected also by *rbcL* and 18S, and *Thalassionema* with *rbcL*, albeit with very low frequencies therefore they are not visible on the figure.

**Figure 5:**
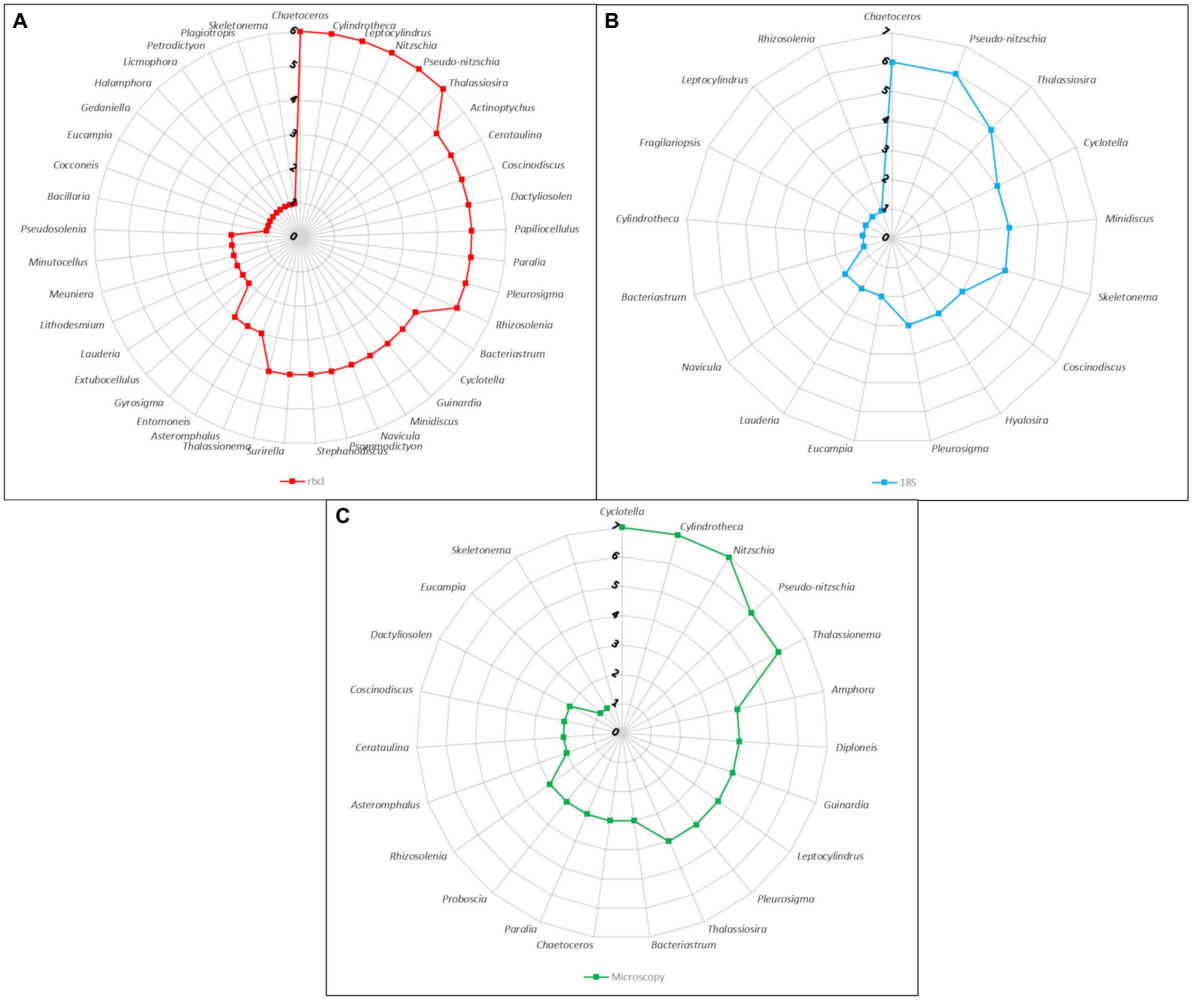
Radar charts of the number of samples harboring genera as identified by different methods. **(A)** *rbcL* data with LULU curation and BLAST assignment. **(B)** 18S-V9 data with NBC50 taxonomy assignment. **(C)** Microscopy count data.

When we consider the number of times a certain genus was recovered across samples we start to see how related the methods actually are (Figure 5). The most common genera such as *Chaetoceros*, *Pseudo-nitzschia*, *Thalassiosira*, *Cyclotella*, *Minidiscus* had similar representation in the metabarcoding data. *Cyclotella* was found in all samples with microscopy, but it is likely that some cells classified as *Cyclotella* actually belonged to other similar genera such as *Actinoptychus* that was also quite common in the *rbcL* data. On the other hand, the occurrence of *Nitzchia* and *Cylindrotheca* was more similar between microscopy and *rbcL*, since the former was not even found by 18S, while the latter was present only in one sample.

**Figure 6:**
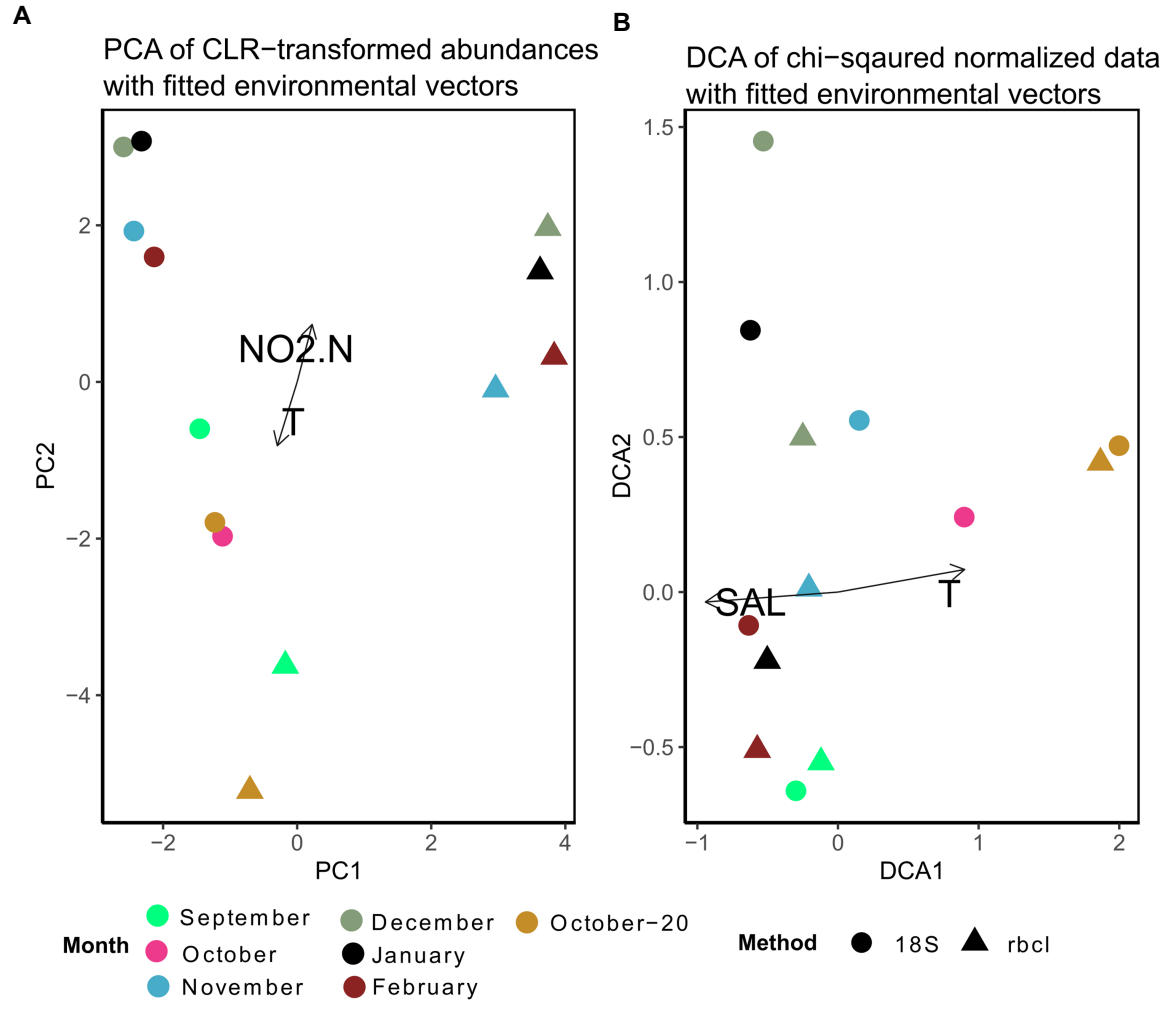
**(A)** Principal component analysis of expected CLR data agglomerated to the genus level for 18S and *rbcL.* Fitted are environmental variables with significant correlations (*p* < 0.05) as determined by the *envfit* function implemented in *vegan*. **(B)** Detrended correspondence analysis based on *χ*^2^–distances of non-transformed data. Ordinations including microscopy samples are shown in Supplementary Figure S4.

For the multivariate comparison of methods, the data were normalized. We show here results of two different normalization procedures, one based on Aitchison distances (Figure 6A) and the other based on *χ*^2^-distances (Figure 6B). Despite the fact, that some samples appeared to be very similar between methods when the data were not normalized (Figure 4), the differences were larger and statistically significant (anosim *R* = 0.51, *p =* 0.001) when the data were normalized with CLR. Even samples that had very similar relative compositions (e.g., October-20) were separated after the transformation. Significant covariates were temperature and nitrite, clearly distinguishing the fall and winter samples. It appears that the winter samples of 18S and *rbcL* were mainly separated by PC1. The highest loadings for this principal component were for *Skeletonema*, *Hyalosira*, *Cylindrotheca*, *Papiliocellulus* and *Cerataulina* (data not shown). Of these genera, only *Skeletonema* and *Cylindrotheca* represented high non-normalized relative abundances, whereas the other three genera were less abundant. *Cerataulina*, *Hyalosira* and *Papiliocellulus* were genera that were uniquely represented by one of the markers. *Hyalosira* only with 18S, and the other two only with *rbcL*. This may point to the fact that the CLR transformation gives more weight to low read abundance taxa that are nevertheless represented in a sample, rather than completely missing.

The ordination stemming from *χ*^2^ normalization resulted in more close clustering of 18S and *rbcL* samples (Figure 6B). Here we present the results of the detrended correspondence analysis (DCA) because the correspondence analysis resulted in a triangular shape of sample scores, which points to high correlation. The structure of the DCA resembled that of Figure 6A with a distribution significantly correlated with temperature and in this case with salinity. Interestingly, samples that appeared to be quite different with the non-normalized data such as September, clustered closely together here. This structure is a consequence of the *χ*^2^ transformation that takes into account not only the change within samples but also individual taxa. It is thus not surprising that genera with the highest loadings in this case were those, that appeared only in one sample in the whole dataset such as *Plagiotropis*, *Halamphora*, *Petrodictyon* and *Extubocellulus*, which were all bunched under “Others” in Figure 4. *Skeletonema* however still had the highest loading with DCA2, similar to the CLR-transformed data. A pairwise comparison of the *χ*^2^ transformed metabarcoding abundance data showed a significant correlation between 18S and *rbcL* data (Mantel statistic based on Pearson’s product–moment correlation: *R* = 0.64, *p* = 0.05), different to the analysis of the CLR transformed data. As you may have noticed, the microscopy data was not included in these ordinations. The microscopy cluster formed a completely separate group with the *χ*^2^ transformed data, except for both October samples, while only the September sample clustered with the metabarcoding data in the CLR transformed dataset (Supplementary Figure S4). The most influential taxa separating metabarcoding and microscopy samples with the CLR data in this analysis were *Minidiscus*, *Thalassionema*, *Nitzschia*, *Cylindrotheca*, and *Skeletonema*, therefore those that had very different relative abundances or were missing from the microscopy data completely. With the *χ*^2^ transformed data, the most influential data in the DCA analysis were different and included *Skeletonema*, *Diploneis*, *Hyalosira*, *Gedaniella*, and *Cocconeis* (data not shown). In addition, no environmental variables were significantly correlated with these ordinations. A pairwise comparison of the *χ*^2^ data did not reveal a significant correlation with the Mantel test (*rbcL*-counts: *R* = 0.54, *p* = 0.13; 18S-counts: *R* = 0.4, *p* = 0.09). This suggested a slightly stronger relationship between 18S and microscopic counts, confirmed by a subsequent coinertia analysis. The inertia was borderline significant when comparing 18S with counts after a Monte Carlo test with 999 replications (*p =* 0.056), whereas the effect was not significant when comparing the *rbcL* and count data (*p =* 0.23).

**Figure 7:**
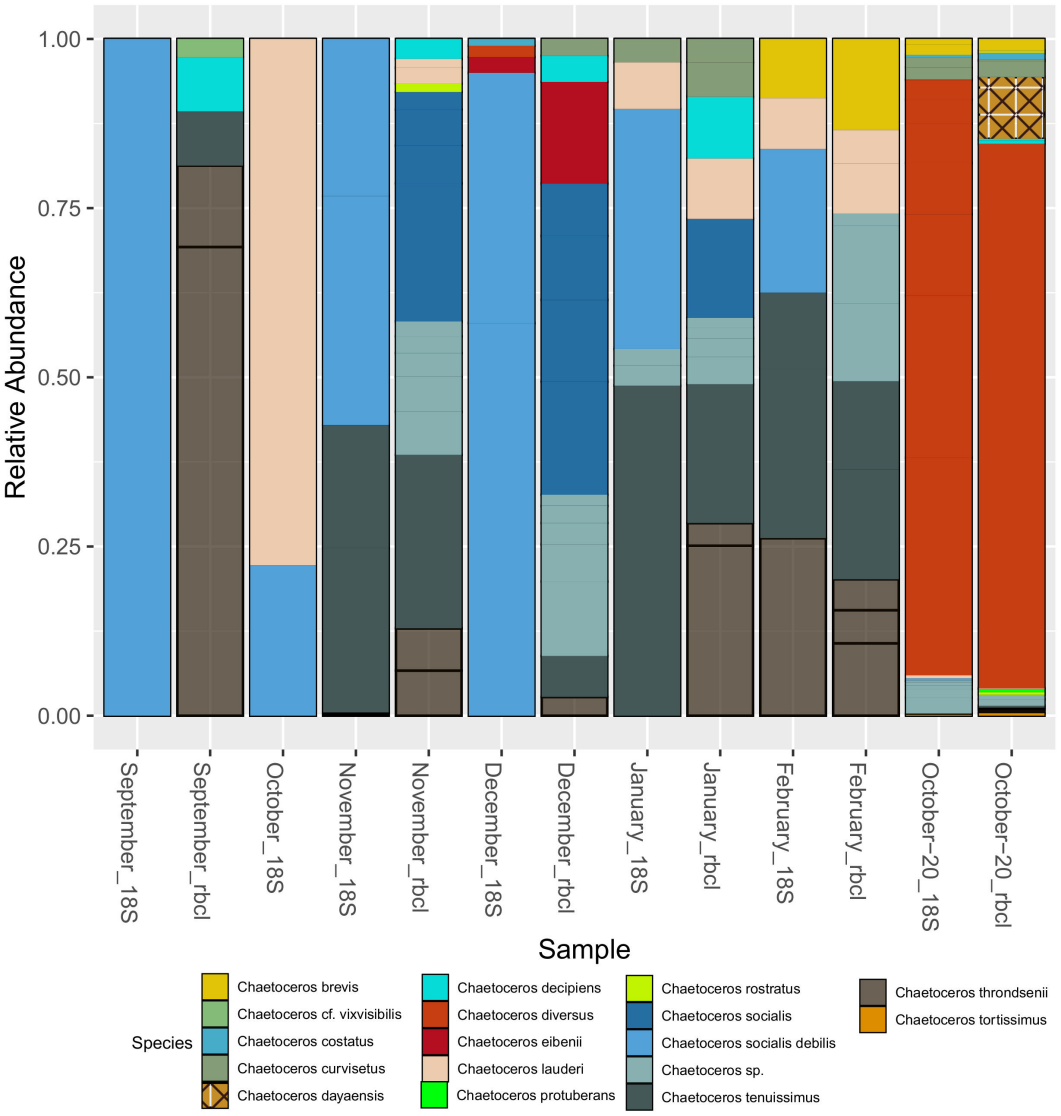
Seasonal distribution of *Chaetoceros* species recovered with 18S-V9 (NBC50) and *rbcL* (LULU+BLAST). The unassigned species in January and February with 18S were assigned to *C. cf. wighmani* with BLAST.

Apart from *Chaetoceros* and *Pseudo-nitzschia*, genera such as *Bacteriastrum* (2), *Papiliocellulus* (2), *Guinardia* (2), *Rhizosolenia* (2), and *Thalassiosira* (7) were represented by more than one species (Supplementary Table S2) both with 18S and *rbcL*. Many of these taxa, especially from the family Cymatosiraceae, were found for the first time in the region, highlighting the power of *rbcL*. A total of 22 new species were identified for the area with *rbcL*_BLAST, including several new genera (Supplementary Table S2). Not surprisingly, given the ambiguity of some genus assignments, identification of diatom species with 18S-V9 appeared to be very unreliable and many species remained unidentified. Most genera were represented by one or two different species. The ability to discriminate species with 18S-V9 was strongly taxon dependent. For example, with *Pseudo-nitzschia*, a common taxon that forms harmful algal blooms, only one species, *P. delicatissima*, was identified with 18S-NBC, and even here the maximum bootstrap support was 77%. This was also evidenced by BLAST, which resulted in several different species assignments with very similar or identical E-scores for *Pseudo-nitzschia* ASVs. *rbcL* on the other hand, identified nine. Contrarily, all reads assigned to *Cyclotella*, for example, were unambiguously assigned to *Cyclotella choctawhatcheeana* by both markers. Where both markers were comparable in terms of species identification was *Chaetoceros* with multiple species identified. This is also why we chose this genus to compare the species composition with that recovered by *rbcL* (Figure 7). Following the results presented above and the fact that NBC50 was based on the curated PR2 database, i.e., more reliable taxonomy, we chose these results for the comparison, even though of 148 diatom ASVs, BLAST identified 49 as *Chaetoceros*, while NBC50 identified only 35. Of these, 31 were identical between the two methods. In total, 10 species were identified with 18S-V9, while *rbcL* identified 16. Microscopic identification of *Chaetoceros* species was qualitative so we can provide only species lists for comparison (Supplementary Table S3). The composition was somewhat comparable between 18S and *rbcL* especially in the winter months and in October-20, similar to the genus data presented above. In September, 18S recovered only *C. socialis debilis*, which was not found by *rbcL* at all. Microscopy also identified *C. cf. vixvisibilis* and *C. decipiens* that were identified by *rbcL*, along with several other species. The two species that were found by 18S in October (*C. lauderi* and *C. socialis*) were also found by microscopy, but the list of October species was far greater for microscopy. The November assemblage was more comparable between 18S and *rbcL* with both *C. tenuissimus* and *C. socialis* recovered by both markers, while *rbcL* recovered some additional species. Interestingly, microscopy did not find any *Chaetoceros* in this month. A similar case was observed in December, where only *C. socialis* was found by microscopy, while the metabarcoding markers recovered more species. In January and February, the metabarcoding assemblages were highly similar, while they differed from the assemblage recovered by microscopy. October-20 showed highly similar assemblages, which were similar to those identified by microscopy. Common species included *C. decipiens*, *C. curvisetus*, *C. lauderi*, *C. rostratus*, *C. socialis*, and *C. tortissimus*. *rbcL* additionally identified *C. dayaensis*, a novel species for the Gulf of Trieste. The obvious difference between metabarcoding and microscopy was the high prevalence of *C. diversus* in metabarcoding data, which was not found at all by microscopy. Another novel species for the Slovenian side of the GoT, *Chaetoceros tenuissimus* was present in almost all samples with significant relative abundance. This species could have been identified as *C. simplex* with microscopy and may be missed in counts due to its inconspicuous morphology. It is worth noting, that particularly *rbcL* identified several additional *Chaetoceros* without formal description, including undescribed but commonly referred to strains (e.g., *Chaetoceros* sp. Na28A1 (also with 18S), *Chaetoceros* sp. Na11C3) which were all grouped to *Chaetoceros* sp. in Figure 7. This shows that for *rbcL* the reference database is underpopulated with several species that have not been barcoded yet, but also some that have not been taxonomically resolved.

### 3.3. *Pseudo-nitzschia rbcL* population structure

**Figure 8:**
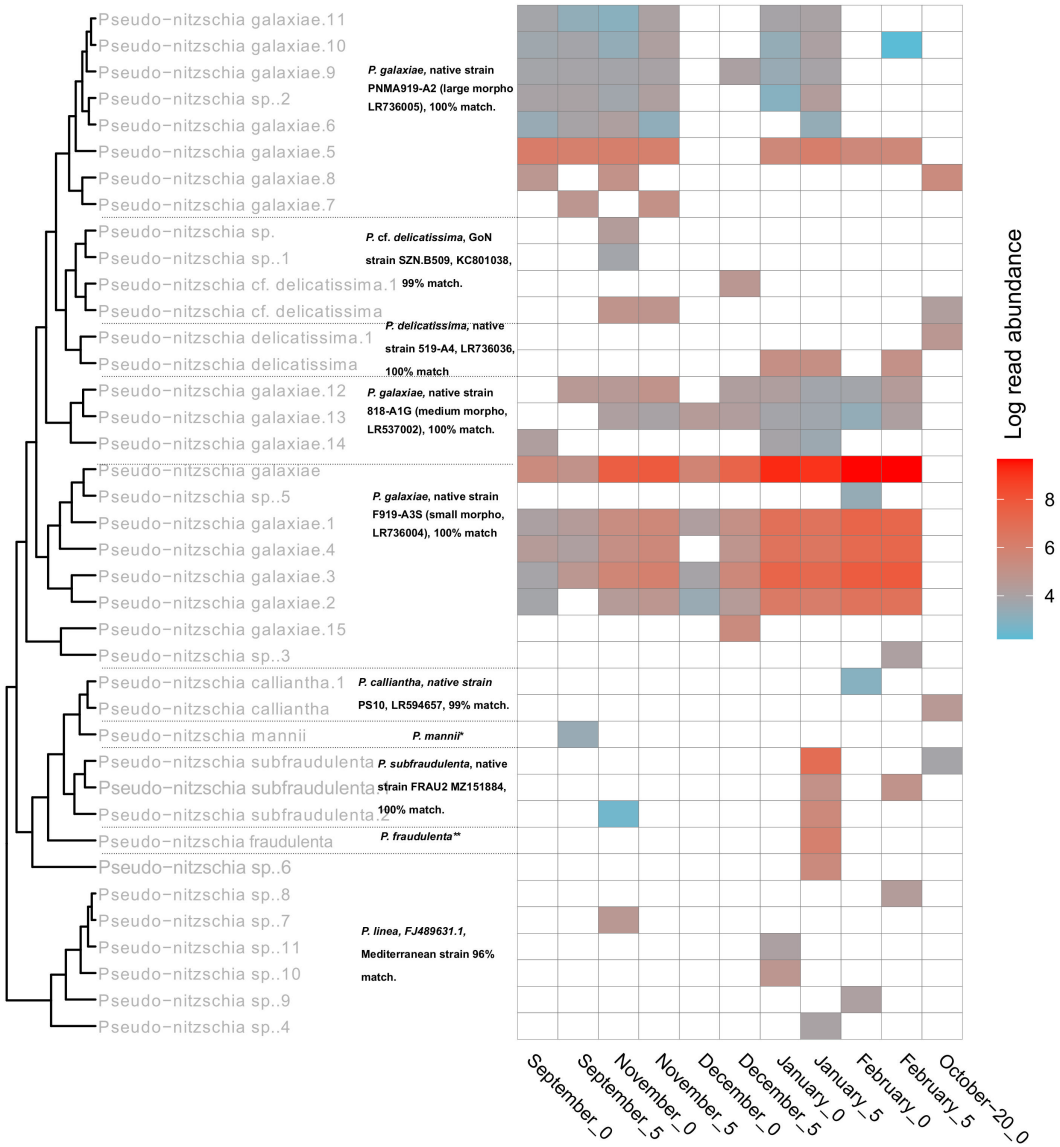
Seasonal succession and phylogeny of different *Pseudo-nitzschia* haplotypes inferred from *rbcL*. Tree labels in gray represent classifications following the NBC50 assignment, labels in black represent top hits of BLAST. The asterixes were used to report top hits in the caption for a clearer representation. *P. mannii, native strain PS9, LR594653. GoN = Gulf of Naples. **P. fraudulenta, native strain 217-A2, LR537014.1.

With *Pseudo-nitzschia*, a heavily studied genus in the area, the NBC50 approach identified 7 species, namely *P. calliantha*, *P. delicatissima*, *P. fraudulenta*, *P. galaxiae*, *P. linea*, *P. mannii*, and *P. subfraudulenta*, while several species-level reads remained unclassified. Three different morphotypes of *P. galaxiae* were also found, which have been shown to be genetically distinct in previous work (Turk Dermastia et al., 2020). Additional species were identified using BLAST. These included *P. delicatissima* and *P. cf. delicatissima*, which represents a different clade within the *P. delicatissima* complex, while *P. linea* was detected only at the 95% identity threshold. The distribution of *Pseudo-nitzschia* species was month and for some taxa also depth dependent (Figure 8). Here LULU-curated data are shown in order to ensure robust representation of the population structure. The most abundant ASVs of strains that were previously isolated and barcoded in the area were identified by BLAST as almost identical to these strains. This was the case for *P. delicatissima*, *P. fraudulenta*, *P. subfraudulenta*, *P. mannii*, all three morphotypes of *P. galaxiae* and *P. calliantha*. *P. galaxiae* was the most dominant species. In particular, the small morphotype was present throughout the sampling period except in October-20, but with a marked increase in February and January. ASVs belonging to the small morphotype were the most abundant *P. galaxiae* ASVs. ASVs of the large morphotype of *P. galaxiae* had increased abundance in September and November compared to the winter months. The medium morphotype had the lowest number of ASVs of the three and a peak amplicon abundance in November. Some species, such as *P. fraudulenta*, *P. subfraudulenta*, and *P. mannii*, showed distinct occurrence patterns, with the former two peaking in January at a distinct depth (5 m), while *P. mannii* occurred only in September. *P. delicatissima* ASVs were present in January, February and October-20, while *P. cf. delicatissima* was present in November and December, thus separated from the *P. delicatissima* ASVs. The reference sequence most similar to these reads was strain SZN-B509, which was classified as *P. cf. delicatissima*, a sister group to *P. arenysensis* but ultrastructurally similar to *P. delicatissima* (Lamari et al., 2013). *P. calliantha* appeared in February in low abundance at 0 m and with a higher abundance in October-20. Lastly, several ASVs classified as *P. linea* with 96% identity occurred sporadically throughout the sampling period.

Most assigned species had multiple haplotypes, the number of which correlated with the number of reads for that species (Pearson *r* = 0.71, *p* < 0.001). Most taxa were represented by more than one haplotype, and dominant haplotypes were also recorded, accounting for the majority of reads, especially in January and February 2020, where very dominant haplotypes of *P. galaxiae* and *P. subfraudulenta* occurred. *P. mannii* and *P. fraudulenta* were represented by only one haplotype.

**Figure 9:**
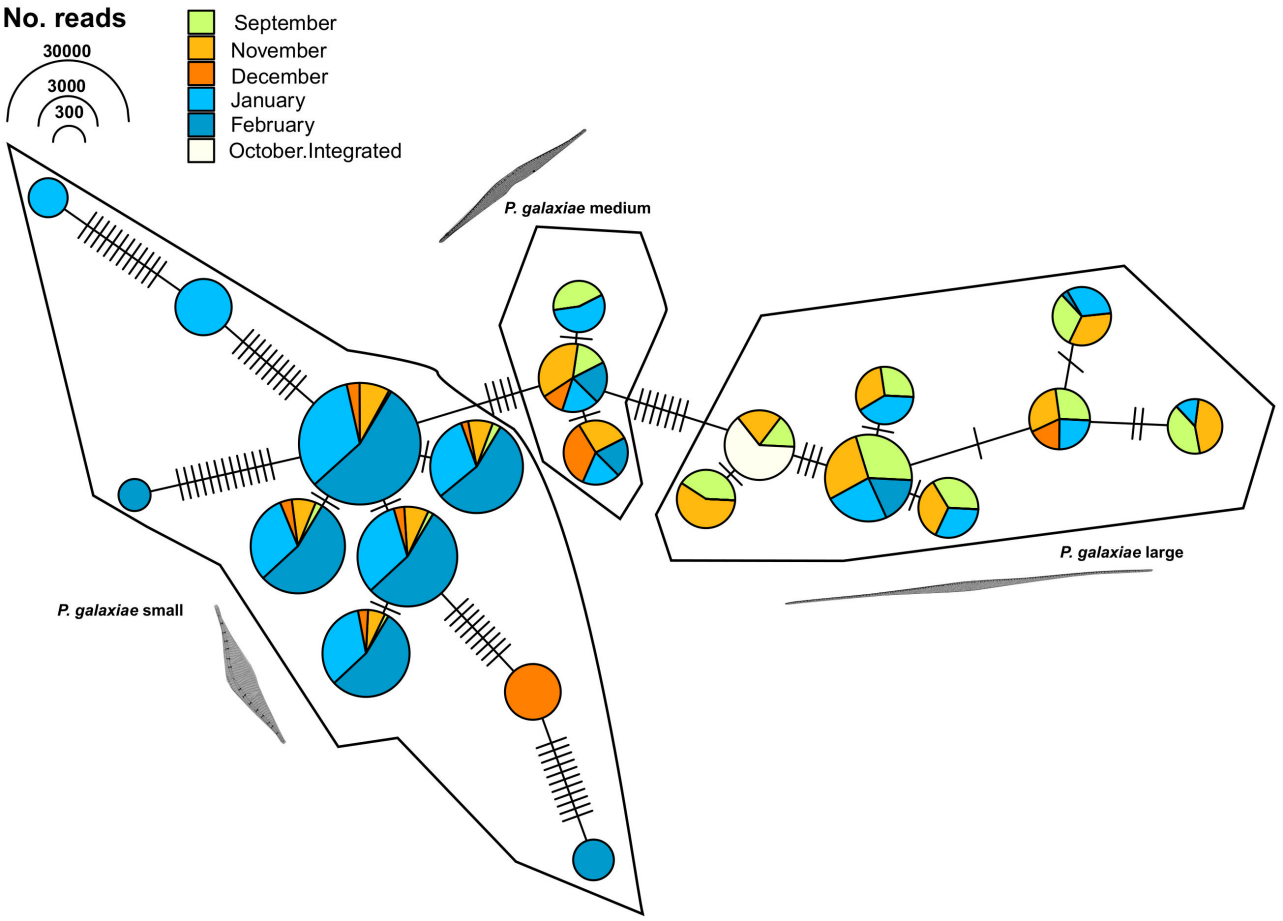
TCS maximum parsimony haplotype network for ASVs classified as *Pseudo-nitzschia galaxiae* by *rbcL* (LULU+BLAST). Three different clusters, corresponding to morphological variants of *P. galaxiae* were recognized. Note that alternative network links are not shown, therefore adjacent haplotypes of different clusters appear to be more related than members of the same cluster, while they are in fact additionally separated for the mutational steps depicted between haplogroups.

To further clarify the suitability of the *rbcL* marker for population genetic studies, we separately analyzed the haplotypes classified as *P. galaxiae* using haplotype network analysis. We see three distinct haplogroups, each corresponding to different morphotypes of *P. galaxiae* (Figure 9). The haplogroups were seasonally well distributed even though the time series did not even encompass the entire year. The large morphotype was most abundant in late summer and fall, the medium represented a transition to winter, and the small morphotype, which was also represented by most of the haplotypes was most abundant in winter. Nevertheless, most haplotypes co-occurred. Haplogroups were likely reproductively isolated as they either represent several sympatric populations that cannot interbreed due to size differences and are already in the process of speciation, or they may already represent different species. Note that Figure 9 shows the maximum parsimony network in which alternative connections are not shown. This may indicate that haplotypes within the same cluster are more distant than those between clusters, which is not the case as the links between clusters show additional mutational steps from the basal state.

## 4. Discussion

### 4.1. Methodological overview

This work presents a comprehensive analysis of the *rbcL* marker for metabarcoding marine diatoms, not only for diversity assessment, but also for population genetics and microevolutionary studies. It is also the first metabarcoding study of planktonic protists in the Gulf of Trieste and also one of the first in the Adriatic Sea, apart from the metabarcoding study in the Venice Lagoon (Armeli Minicante et al., 2020). The study, conducted with a half-year series of samples collected monthly, was short but provided insight into the hidden diversity and community patterns unknown in more than 30 years of continuous microscopic monitoring.

The choice of markers in our study, the 18S-V9 region and *rbcL*, was based on previous practice of metabarcoding marine plankton and novelty. 18S-V9 is a well-established eukaryotic marker that has been used in many metabarcoding experiments and campaigns, including the global Tara Oceans cruise and the annual Ocean Sampling Day (De Vargas et al., 2015; Bradley et al., 2016; Malviya et al., 2016; Piredda et al., 2017; Stefanni et al., 2018; Tragin et al., 2018). In this sense, the 18S-V9 data are useful for comparison with other published metabarcoding surveys and the present *rbcL* assessment, while they lack the perhaps desired species-specific robustness. This was also evident from our results, with the 18S marker generally underperforming compared to *rbcL* in both diatom ASVs and recovery of classified diatom taxa. This is likely due to two factors, the first being greater sequencing depth and the use of diatom targeted primers with *rbcL*, exemplified by species accumulation curves (Supplementary Figure S1). The second factor is the fact that the *rbcL* region used in this study is in fact more variable than 18S-V9. However, this observation was taxon dependent. For example, in the genus *Chaetoceros*, species recoveries were similar between the two methods in certain months, although with different relative abundances. On the other hand, the number of *Pseudo-nitzschia* sequences recovered with 18S was very low, probably also due to the incompleteness of the reference database (Piredda et al., 2017). This was illustrated by the two different taxonomic classification approaches, with NBC50 correctly recognizing winter-blooming *Pseudo-nitzschia galaxiae* and classifying reads as *Pseudo-nitzschia* spp. while BLAST assigned these sequences to *Fragilariopsis kerguelensis*. The lack of a reference for *P. galaxiae* 18S-V9 has already been pointed out by Piredda et al. (2017). In this sense, 18S-V4 might be a more appropriate marker, and we acknowledge that the assessment performed in this work would benefit from 18S-V4 data. On the other hand, barcoding effort was far greater with 18S than with *rbcL* and many diatom taxa lack their reference *rbcL* barcodes (Guo et al., 2015). Our study also shows that 18S data resulted in less unassigned ASVs than *rbcL* although the proportions were still quite similar. Furthermore, taxonomic assignment of *rbcL* ASVs with BLAST resulted in less unassigned amplicons compared to the NBC50 approach that used a curated database, with the caveat of the assignments being less reliable.

Traditionally, the phytoplankton community is determined by counting phytoplankton cells under a light microscope. This method is still widely used and the counts have been the gold standard in environmental assessments by government agencies and research institutes for decades. However, the method has its drawbacks, namely dependence on a trained observer, i.e., the taxonomist who counts the cells, inability to distinguish cryptic or inconspicuous taxa, insensitivity to rare taxa, and low sample throughput. The latter may not be important when the number of examined samples is small, but becomes critical when sampling is intensified in time and space. In addition, phytoplankton count data can be valuable in their own right, but they are rarely accompanied by other important cellular characteristics such as biomass that could be more ecologically informative (Godhe et al., 2008; Santi et al., 2021). Thus, it has been shown in recent years that identification and counting of cells under the microscope is not sufficient. Metabarcoding solves all the above problems, but also brings some problems of its own. First, it is PCR-dependent. PCR may preferentially amplify some targets while not amplifying others or amplifying them at a lower rate (Kelly et al., 2019). Second, taxonomic assignment relies on reference databases that do not cover all genetic diversity because most organisms have never been cultured and sequenced (Weigand et al., 2019). Therefore, assignment may be ambiguous or, in some cases, not possible at all, as it is very likely that metabarcoding in a new environment will yield a large number of new sequences. A conceptual problem also arises in translating sequence data into taxonomic richness at the species level. What is a genetic species and up to what point are dissimilar sequences considered a species?

Finally, there are no simple quantification strategies to convert abundance from sequence data into ecologically meaningful abundance, especially given the bias mentioned earlier. Sequencing machines have pre-defined upper thresholds of obtained reads and thus more abundant (or more amplified sequences) can take up a large proportion of the designated sequencing space on chips, artificially skewing the signal (Gloor et al., 2017). Here we approach this by treating the data as compositional, as proposed by Gloor et al. (2017) using the Aitchison CLR transformations. The crucial advantage of the CLR normalization is that it takes into account the exponential differences between more abundant and less abundant taxa that occur after PCR (signal amplification) but also because it does not remove data. This is important for the comparison of two different markers, each of which had a different sequencing depth and priming properties. Furthermore, the sampling was performed in a heterogeneous and fast-changing environment for which the existing diversity is well known from years of biological observation, mainly through microscopy. Therefore, we did not want to omit genera or species recovered with metabarcoding through rarefaction, which is the more standard yet widely disputed (McMurdie and Holmes, 2014) method of normalization. Authors such as Cameron et al. (2021) suggest the limitations of classic rarefaction could be tackled by repetitive rarefaction, however this still preserves the amplification bias. We believe such an approach could be beneficial in less known and understudied systems such as microbiomes, but for comparative purposes with traditional methodology of higher organisms, the benefits of such an approach are less clear. On the other hand, we stress that no normalization procedures is perfect and each has its associated biases. With CLR this is clearly the means of treating zeros, either with pseudocounts such as in our case, or with different imputation methods that estimate the actual values from prior distributions (e.g., Bayesian-multiplicative replacement).

Still, the resulting transformed data is hard to interpret and compare with microscopy, particularly in terms of abundance of different genera. A better way to compare the results of morphological and genetic methods might therefore be data on the biomass or biovolume of different phytoplankton groups (Olenina, 2006; Godhe et al., 2008; Leonilde et al., 2017), although these data are rarely obtained. There is a clear relationship between cell volume and rDNA content that has been demonstrated in dinoflagellates (Prokopowich et al., 2003). They are known to be over-represented in metabarcoding datasets, with a high proportion of unclassified reads below the phylum level (Piredda et al., 2017; Santi et al., 2021). Dinoflagellate rDNA from the 18S-V9 metabarcoding had previously been analyzed for agreement with true abundances of organisms in mock communities, and the percentages found were quite variable (Guo et al., 2016). The authors found that the actively translated actin gene is a better representative of the community. In our study, the *rbcL* gene was sequenced to enable a different type of identification and quantification strategy, although the relationship between biovolume and *rbcL* copy number is believed to be similar to the relationship between biovolume and 18S copy number– it varies significantly from species to species (Godhe et al., 2008; Vasselon et al., 2018). Vasselon et al. (2018) proposed correction factors based on biovolume that significantly improved abundance estimates of freshwater diatoms. In the case of natural populations this is difficult to apply, since even members of the same species (e.g., *Pseudo-nitzschia galaxiae*) can vary significantly in this characteristic. We thus opted not to apply these correction factors to the *rbcL* data, even though they could improve the correlation with microscopic data. This fine-tuning was not the purpose of this study, but we acknowledge it can be applied if accurate abundance estimates from metabarcoding are sought.

The use of diatom-specific *rbcL* primers has limited us to diatoms, although many “phytoplankton taxa” such as different dinoflagellates do not actually have chloroplasts and therefore a different type of bias would be introduced if universal *rbcL* primers were used. Another problem arising from next-generation sequencing platforms and subsequent bioinformatics analysis is the inflation of operational taxonomic units (OTUs) due to errors in sequencing data generation. OTUs are consensus sequences from multiple reads that differ up to an arbitrary threshold set at 97% in most studies. They are used to infer the taxonomic identity of sequences and to measure the diversity of the communities under study. Alternatively, amplicon sequence variants (ASVs) can be used, such as is in this study so that maximum genetic information was retained. ASVs are sequences that are 100% different and have been obtained from metabarcoding, but have been informatically corrected for errors introduced by the sequencing platform (denoising). Algorithms such as *dada2* are capable of such correction, but the efficiency is still questionable (Nearing et al., 2018). For the most part, sequencing errors do not pose a problem for diversity estimation, as erroneous sequences do not usually occur in large numbers (Frøslev et al., 2017; Turon et al., 2020). However, when intraspecific or haplotypic diversity is desired, these errors can artificially inflate the number of haplotypes and lead to erroneous results. In this study, we have applied the LULU curation algorithm (Frøslev et al., 2017) in order to minimize noise in the data. The noise was drastically decreased as was evidenced by a substantial decrease in ASV number as was in the calculations of the entropy ratios, which could be obtained as *rbcL* is a coding gene. The obtained haplotypes used in the analysis of *Pseudo-nitzschia* population structure are thus robust as they are highly similar to haplotypes of strains previously isolated in the region (Turk Dermastia et al., 2020, 2022). To our knowledge, this is the first study to examine the performance of the *rbcL* marker for metabarcoding of marine diatoms. *rbcL* has already been used for metabarcoding of freshwater diatoms (Vasselon et al., 2017a). Our study yielded comparable results to 18S-V9, but with higher resolution and detail. We acknowledge that using diatom-specific 18S primers or greatly increased sequencing depth with 18S would benefit the final verdict on whether *rbcL* is superior to either region of 18S for diatom metabarcoding.

### 4.2. Diatom assemblages revealed by metabarcoding

The composition of the diatom assemblage determined by 18S-V9 and *rbcL* metabarcoding agreed quite well with long-term patterns in this region described from phytoplankton microscopy counts, while providing new insights into the structure of the assemblage. Diatom blooms in autumn are expected in the northern Adriatic (Mozetič et al., 1998; Cabrini et al., 2012; Mozetič et al., 2012; Cerino et al., 2019). Autumn and early winter, at least in the Italian part of the Gulf of Trieste, are characterized by low diatom abundance and greater diversity (Cabrini et al., 2012; Cerino et al., 2019), while in the Slovenian part autumn blooms are more abundant (Mozetič et al., 2012; Vascotto et al., 2021). In this study, microscopy followed the long-term trends and showed a peak in diatom cells in both October samples. With the normalized metabarcoding data a peak relative to other samples was observed only in October-20 (Figure 2). It is worth noting here that the progression of 18S and *rbcL* was similar, even though *rbcL* detected predominantely diatom reads, whereas 18S detected the entire eukaryotic community. This suggest that the normalization procedure with CLR does a good job at approximating the true composition.

Amplicon sequencing is a suitable approach for comparing diversity estimates with other methods. While *rbcL* recovered much more ASVs, which was also reflected in a greater number of species and genera, the Simpson’s diversity and Shannon’s diversity indexes were comparable between methods. Even though estimates for individual samples were different, the TukeyHSD test did not show significant differences between the methods and taxonomic approaches. This result is encouraging since it suggests that *rbcL* can be reliably used as an estimator of diatom diversity, while providing a much greater resolution when used as it was in this study.

At first glance, the qualitative comparison of genus and species recovery between metabarcoding and microscopy showed large differences, while the two metabarcoding markers were much more comparable. This was confirmed also with the statistical tests we applied, which also indicated that 18S and microscopy were more correlated. A reason for this could be that due to deeper sequencing and greater variability, *rbcL* recovered more “rare” diversity which is difficult to find also by microscopy, producing differences in datasets. The other reason is hinted in samples where the abundance of diatoms determined with microscopy was low such as in the winter samples. Here the discrepancies were high because *rbcL* amplified the already rare diatom signal which likely skewed the composition. The non-normalized relative abundance of taxa found only by *rbcL* was generally low, but following normalization these differences started to influence the changes between the compositions obtained by *rbcL* and 18S leading to the observed differences in sample clustering (Figure 6). As already hinted in the results, the most influential taxa where in fact those that had either large differences in relative abundances or were entirely absent from one or the other dataset. On the other hand, in certain months such as September, where the number of 18S diatom ASVs was unusually low, microscopy and *rbcL* were more similar. The only month where all methods really concurred was October-20, especially when we consider the correspondence analysis. We must be cautious with these samples however, since they were obtained with a phytoplankton net of 20 μm mesh size, thus favoring larger diatoms. These samples thus inherently miss the smaller diatom fraction, which could hardly be identified by microscopy, leading to higher similarity. Interestingly, the Simpson’s Diversity index obtained for this month was actually the most different from 18S and *rbcL*. We used different normalization procedures (CLR and *χ*^2^) to perform the β-analysis. The CLR method normalized the read numbers sample-wise while the *χ*^2^ method normalized both taxon and sample wise. Technically, the sample wise logarithmic normalization is more appropriate for high-throughput sequencing data, since amplification bias is expressed exponentially and is related to the individual sample (Gloor et al., 2017). Some similar patterns between the two approaches emerged, but the correspondence analysis using *χ*^2^ transformed data resulted in more common structure between 18S and *rbcL*. In both cases, the seasonal clustering related to temperature was evident, while the most influential taxa, except for *Skeletonema* differed. The CLR approach clearly gives a lot of weight to rare but present taxa, whereas the *χ*^2^ is less sensitive to the absence of a particular genus in a certain sample. This is because the expected abundance of a rare taxon from the *χ*^2^ distribution will still be low, whereas in the CLR approach the normalization could increase the importance of rare taxa, accounting for PCR and sequencing bias (Gloor et al., 2016).

Several genera and species were found for the first time in the area, and several of them had significant relative abundances. Examples include *Minidiscus* and certain *Thalassiosira* species. In particular, *Minidiscus* is globally overlooked in phytoplankton counts, while its biomass and abundance potential has been captured in other metabarcoding studies (Leblanc et al., 2018; Arsenieff et al., 2020). Our work adds to these findings, as *Minidiscus* was never recorded by microscopy, although it accounted for up to a quarter of the diatom assemblage in December. Still, this represented small relative numbers within the entire phytoplankton community, as diatom numbers in December were the lowest. In a recent study of Adriatic ports, *Minidiscus* was tentatively identified only in the port of Trieste among 12 studied ports across the entire Adriatic basin (Mozetič et al., 2019). We confirm the presence of this genus here with the identification of *Minidiscus trioculatus*. 18S recovered a relatively small number of genera, comparable to the number of microscopy. Some common genera such as *Thalassionema* and *Nitzschia*, identified under the microscope and with *rbcL*, were absent among the taxa found. However, we must keep in mind that 18S sequencing was not tailored to diatoms. Therefore, a large proportion of the ASVs in the 18S dataset belonged to taxa that were not diatoms, whereas the majority of *rbcL* were diatoms. Among identified species, this discrepancy between markers was even greater, as we have shown using *Pseudo-nitzschia* and *Chaetoceros* as examples. The *rbcL* marker provided much higher resolution when considering species or even genera, with 22 new species in the study area, including 6 new genera. Since metabarcoding is an eDNA-based method, the source of amplicons could also be dead cells, cell fragments, resting stages and solubilized nucleic acids (Corinaldesi et al., 2008). This could be especially true for organelle-encoded genes, as organelle membranes provide additional protection against degradation. We speculate that at least some of the assigned taxa, such as *Pseudosolenia calcar-avis*, may be derived from such sources, as it is an extremely large diatom that is unlikely to be overlooked during microscopy, but was not found in the analyzed microscopy samples. Both data sets (18S and *rbcL*) contained a very high proportion of putative diatom sequences without genus or even species assignment. This is a common phenomenon in metabarcoding datasets obtained in novel regions, but such patterns are also common in areas with extensive sampling (Malviya et al., 2016; Piredda et al., 2018). Malviya et al. (2016) have shown that in global datasets 30–80% of all reads are unassigned at the genus level, with the percentage strongly dependent on the size fraction of diatoms, with smaller diatoms having larger percentages of unidentified amplicons. This is exemplified with our October-20 samples, obtained with a 20 μm phytoplankton net, and where the proportion of unassigned genera was in fact the smallest. The precentages of unassigned reads reported by Malviya et al. (2016) are comparable to both our 18S and *rbcL* data, with *rbcL* generally showing slightly higher unassigned rates. For *rbcL*, there is certainly also a lack of reference sequences, as the sequencing effort for cultured phytoplankton was much lower compared to 18S (Guo et al., 2015). Therefore, the larger proportion of unassigned reads is not surprising. On the other hand, it was surprising that the proportion of unassigned taxa in microscopy was very similar to that in metabarcoding, suggesting that taxa that have not yet been cultured can be detected by microscopy and selectively cultured to increase our knowledge of marine diatoms and improve reference databases.

Results of the comparative study are similar to Malviya et al. (2016), who showed that correlations between metabarcoding and microscopy exist but are not initially obvious. The discriminatory power of light microscopy is known to be much lower than that of metabarcoding. Metabarcoding recovered nearly twice as many taxa than microscopy in freshwater diatoms, for example (Zimmermann et al., 2015). Indeed, the main cause of the differences between the morphological and metabarcoding datasets were small diatom taxa such as *Minidiscus trioculatus* and *Thalassiosira* (several species), but also *Pseudo-nitzschia galaxiae*, which were largely overlooked by microscopy. Another obvious source of divergence between phytoplankton counts and metabarcoding data was the high number of *Cylindrotheca* counts in winter, which were replaced by *Pseudo-nitzschia* reads in the metabarcoding data. Since most of these reads belonged to *P. galaxiae*, we hypothesize that these differences are actually due to misclassification of *P. galaxiae* as *Cylindrotheca closterium* because the cells look very similar (Turk Dermastia et al., 2020). Many taxa that appeared in the *rbcL* dataset were benthic (e.g., *Psammodictyon*, *Cocconeis*), which is not surprising since most known diatom species are benthic and their presence in planktonic datasets with low reads has also been demonstrated in other diatom metabarcoding studies (Piredda et al., 2018). In addition, *Amphora*, another benthic genus has also been detected with microscopy. Interestingly, some of these genera including *Minidiscus*, *Cylindrotheca*, *Cocconeis*, *Amphora*, were among the most influential for the clustering of microscopy and mentabarcoding samples in the performed multivariate analyzes.

As for species data, we focused on *Chaetoceros* and *Pseudo-nitzschia*, as these genera have already been well studied (Turk Dermastia et al., 2020; Janja Francé, personal communication), with the former being even the most diverse diatom genus of the nearshore waters of the Adriatic (Mozetič et al., 2019). The number of *Chaetoceros* species was similar for both methods, although the *rbcL* marker provided higher resolution and more details. Most of the species found have been previously recorded in the northern Adriatic (Bosak et al., 2009; Godrijan et al., 2013), with the exception of *C. dayaensis*, which was identified by *rbcL*. This species was described in Chinese tropical waters (Li et al., 2015). Some of the ASVs classified as *C. dayaensis* had 100% identical sequences to published ones, while others showed only 97% sequence similarity, suggesting there may be other related species present that have not been barcoded yet. The bloom of *C. socialis* found with 18S in September was also in accordance with previous studies investigating this genus (Bosak et al., 2009; Godrijan et al., 2013). In the records of the Slovenian harmful algal bloom (HAB) monitoring program, *C. socialis* was also found in this period (Janja Francé, personal communication, Supplementary Table S3). With *rbcL*, maximum abundance occurred in November and December, similar to one of the published studies (Godrijan et al., 2013). *C. throndsenii* and *C. tenuissimus* were present throughout the study period and were recovered by both markers. They also represented the majority of *Chaetoceros* amplicons. The other recovered *Chaetoceros* species had restricted occurrence windows that were very similar to the published data. One group of reads in January and February with *rbcL* could not be identified to species level. This may be *C. cf. wighamii*, which was recognized by 18S but only with BLAST, which was not shown in Figure 6. *rbcL* reference sequences for this species were not available at the time of analysis. This species was previously found in the region by microscopy but it is a relatively new recognized taxon. The microscopic phytoplankton monitoring program in the Slovenian part of the Gulf of Trieste does not distinguish between many *Chaetoceros* species. On the other hand, many species are recognized but not quantified as part of the HAB monitoring program. This data was used on our case for comparison. Many more *Chaetoceros* species were identified by microscopy in certain months compared particularly to 18S metabarcodes. The assemblage compositions were comparable but not the same. This can be explained by the unsystematic way in which the presence of species was detected under the microscope, but possibly also due to misclassification. For example, *C. tenuissimus*, a very common representative in the metabarcoding data is not found in microscopy but may instead be classified as *C. simplex*. With *rbcL* the lack of reference sequences was really evident, since there was a large share of ASVs that were classified as *Chaetoceros* sp., while with 18S there were only a handful. This is thus perhaps the largest burden of *rbcL* metabarcoding at present, especially for genera with complex taxonomies and hundreds of species such as *Chaetoceros*.

Diatoms of the genus *Pseudo-nitzschia* were detected with both the 18S and *rbcL* markers. In terms of number of species identified, *rbcL* performed much better, but at the genus level the markers were comparable. 18S was only able to discriminate *P. delicatissima*, although given the discriminatory power of 18S-V9 and the fact that *P. delicatissima* was represented by only a handful of *rbcL* reads, we assume that these sequences actually belonged to another species, possibly *P. galaxiae*. *rbcL* detected 8 different species (*P. calliantha*, *P. delicatissima*, *P. cf. delicatissima*, *P. galaxiae*, *P. linea*, *P. mannii*, *P. fraudulenta*, *P. subfraudulenta*). Most were previously found in the GoT with very similar seasonal distributions determined by a combination of environmental and cultural techniques (Turk Dermastia et al., 2020). Of these a new confirmation for the area was *P. linea*, although it is thought to be overlooked in microscopic monitoring due to its small size and epiphytic nature, usually associated with *Chaetoceros* (Lundholm et al., 2002, 2012; Ruggiero et al., 2015). ASVs classified as this species had lower similarity to published sequences than the other *Pseudo-nitzschia* ASVs. This is probably also due to the lack of reliable reference sequences. The ASVs belonging to this species occurred sporadically, with more reads present in winter. *P. linea* represents a new species for the area. *P. linea* is known to occur in the Mediterranean and has been found mainly in the winter months (Quijano-Scheggia et al., 2010; Ruggiero et al., 2015). Among the species found, there are also several clusters of sequences identified as *P. galaxiae*, representing different morphological types (Figure 9). The phylogeny of *P. galaxiae* is complex and it has been demonstrated several times that it is probably a species complex in which the morphological types are genetically distinct, which could also be related to their toxicity (Ruggiero et al., 2015; Turk Dermastia et al., 2020, 2022). The strains of *P. galaxiae* show great diversity, which was also confirmed by our correlation analysis between the number of haplotypes and ASV frequency. Another novel species in the study area we identified is *P. cf. delicatissima*, which was also time-separated from its sister species *P. delicatissima*. This taxon was previously described from the Gulf of Naples, where it showed a very similar pattern of seasonal occurrence, appearing from September to December as in our data (Lamari et al., 2013; Ruggiero et al., 2015). We could not detect *P. pungens* and *P. multistriata* in our metabarcodes, although these species are known to occur here (Turk Dermastia et al., 2020). However, in recent years, the former species was no longer found in phytoplankton monitoring samples, while the other is very rare (Janja Francé, personal communication). Our study shows, that with complete reference databases *rbcL* metabarcoding can provide near strain-level resolution of the assemblage. This is important for monitoring HAB species. *Pseudo-nitzschia galaxiae* is known to be toxic in this area (Turk Dermastia et al., 2022), with the toxicity being strain dependent. Our study provided insight into the occurrence and frequency of different haplotypes (strains), although this strategy is still too time-consuming because actions to manage HABs must be taken in near real time. It is however, a valuable tool to identify novel strains, species or even genera in a particular study site, that are missed by microscopy or are even new to science.

Two of the most surprising results are the dominance of *P. galaxiae* ASVs in this study and the rarity of *P. calliantha*. The high number of *P. galaxiae* ASVs in January and February was surprising mainly because *Pseudo-nitzschia* is rarely detected in high numbers by cell counting in these months (Turk Dermastia et al., 2020; Vascotto et al., 2021), including cell counts in the year 2020 when samples were collected for metabarcoding. The tiny size of the small *P. galaxiae* morphotype could make the counts very unreliable, first because of the low detection rate and second because of misclassification. One cause of this misclassification is already apparent in our data, because there was a disproportionately large difference in the relative abundance of *Cylindrotheca* and *Pseudo-nitzschia* obtained by microscopy and metabarcoding in these months (Figure 4). *Pseudo-nitzschia galaxiae* is easily misinterpreted as *Cylindrotheca closterium*, especially in fixed samples. This is therefore a clear example of how biased long-term microscopic data sets can be and why it is critical to introduce barcoding techniques into protist monitoring programs (Stern et al., 2018). As noted earlier, another cause of this discrepancy could again be signal amplification by metabarcoding, since the abundance of diatoms in winter months was low. This should at least partly be addressed by data normalization, which in fact diminished the influence of *Pseudo-nitzschia* but the influence of *Cylindrotheca* remained high. The rarity of *P. calliantha* was surprising, as it was one of the most abundant species in previous studies (Turk Dermastia et al., 2020) and is usually present throughout the year. It is easily confused with *P. mannii* under light microscopy, although previous studies also confirmed its presence using genetic methods as well.

### 4.3. *Rbcl* metabarcoding as a population-genetics tool

Lastly, we discuss the potential of *rbcL* metabarcoding to draw meaningful conclusions about population structure and microevolution. We focused on two widely studied groups, and in the case of *Pseudo-nitzschia galaxiae*, one species for which speciation hypotheses exist (Ruggiero et al., 2015, 2022; Turk Dermastia et al., 2022). When using metabarcoding as a population genetics tool, caution must be exercised with sequencing technology and ASV calling algorithms, which are not error-prone and therefore can artificially inflate haplotype composition (Nearing et al., 2018; Turon et al., 2020). However, when function and speciation are of interest, higher resolution can lead to discriminating taxa based on functionally irrelevant variation and decreasing predictive power for observing environmental and biological covariation (Needham et al., 2017). Thus, a balance must be struck. Several pipelines have been developed to address this problem (Frøslev et al., 2017; Turon et al., 2020; Kleine Bardenhorst et al., 2022). The simplest way to detect errors is to use coding genes and translate the obtained sequences to identify stop codons. In our case, *rbcL* performs this function well, and our haplotypes were all screened for translation errors. Next, we can proceed with OTU picking, although this inevitably reduces the number of true haplotypes. Post-clustering methods can also be used (Frøslev et al., 2017; Turon et al., 2020), which were used in this study to avoid OTU-picking.

In our case, *P. galaxiae* haplotypes clearly showed time-separation that was also related to the inferred morphologies of these haplotypes. The morphological assignments were based on the minimal E-scores in BLAST, which matched the cultured specimens from our culture collection with analyzed morphologies (Turk Dermastia et al., 2020). The most common haplotypes showed identical or near-identical sequences to these strains. This is to be expected as it is statistically very likely that the most common haplotype will be isolated if the sampling effort is not very large, which was not the case in our case as we isolated only 6 strains (Turk Dermastia et al., 2020). The dominant haplotype was usually accompanied by less frequent but still abundant auxiliary haplotypes. Marker genes say nothing about the abundance of evolutionary processes taking place at the genome level, but they can be informative enough (Kashtan et al., 2014). There are many factors that drive these microevolutionary processes. The simplest are mitotic division errors, followed by clonal expansion and bottlenecks that follow blooming periods (Ruggiero et al., 2018). As in classical evolutionary theory, some of these mutations provide an advantage to the new haplotype. For example, it has been shown that virus infection can drive haplotypic differentiation of phytoplankton and that host ASV variation is relevant for protection against the virus (Needham et al., 2017). The existence of *Pseudo-nitzschia* viruses has been demonstrated (Carlson et al., 2016) and also recently isolated from the GoT (unpublished data), while strain specificity for viral infections has also been demonstrated and is common in diatom viruses (Nagasaki et al., 2004). Another commonly observed pattern in metabarcoding data is that more common taxa tend to be more microdiverse (Needham et al., 2017; Ruggiero et al., 2022). In our case, this was true for both *Pseudo-nitzschia* and *Chaetoceros*, with the number of haplotypes positively correlated with haplotype reads. Recently, a study on the haplotypic composition of *Pseudo-nitzschia* 18S-V4 in the Gulf of Naples was conducted (Ruggiero et al., 2022). In this study, no curation of ASV tables was performed, so the haplotypic data sets are likely inflated, but we can see that very similar patterns emerged compared to our *rbcL* analysis. They also identified three clusters of *P. galaxiae* with different seasonal patterns and a very large number of haplotypes. On the other hand, *P. mannii* and *P. fraudulenta* were the least diverse species, similar to our study. The second most abundant species in our study, *P. subfraudulenta*, also had a larger number of haplotypes, while this species was not found in the Gulf of Naples.

In conclusion, we have shown that *rbcL* is a suitable marker for both assessment of diatom assemblages and eDNA-based population studies. This study will serve as a basis for future efforts to bring eDNA-based monitoring into the mainstream as it demonstrates that improved as well as complementary data to traditional monitoring can be obtained. We acknowledge that this study is burdened by a short time-series, while it could benefit from additional sequencing efforts using 18S diatom specific markers, and other regions of the 18S gene. However, due to remaining caveats, it is still better to use a combination of approaches to assess diatom assemblages. This way accurate and reproducible datasets can be obtained, which can serve as a basis for further studies that rely on accurate assessment of diversity. This is important because diatoms contribute immensely to the ocean’s primary production, carbon and silicon budgets. These processes are dependent on the diversity and composition of diatom assemblages (Tréguer et al., 2018), therefore accurate diversity assessments are crucial.

## Data availability statement

The datasets presented in this study can be found in online repositories. The names of the repository/repositories and accession number(s) can be found in the article/Supplementary material.

## Author contributions

TT has conducted the sampling, analysis and has written the draft of the manuscript. IV has contributed to the diversity analysis and provided comments to the manuscript. JF has conducted the analysis of microscopy samples and has provided comments and insight on the manuscript. DS has contributed to the bioinformatic analysis. PM has supervised the study and provided comments and insight on the manuscript. All authors contributed to the article and approved the submitted version.

## Funding

This work was conducted with the help of RI-SI-2 LifeWatch (Operational Program for the Implementation of the EU Cohesion Policy in the period 2014–2020, Development of Research Infrastructure for International Competition of Slovenian Development of Research infrastructure area–RI-SI, European Regional Development Fund, Republic of Slovenia Ministry of Education, Science, and Sport). The authors acknowledge the financial support from the Slovenian Research Agency (ARRS) research core funding No. P1-0237. DS was additionally supported by core funding No. P1-0255 and research project No. J1-3015. TT was supported by ARRS program for young researchers, in accordance with the agreement on (co) financing research activities.

## Conflict of interest

The authors declare that the research was conducted in the absence of any commercial or financial relationships that could be construed as a potential conflict of interest.

## Publisher’s note

All claims expressed in this article are solely those of the authors and do not necessarily represent those of their affiliated organizations, or those of the publisher, the editors and the reviewers. Any product that may be evaluated in this article, or claim that may be made by its manufacturer, is not guaranteed or endorsed by the publisher.
