# Corrigendum: Evaluation of the *rbcL* marker for metabarcoding of marine diatoms and inference of population structure of selected genera

**DOI:** 10.3389/fmicb.2023.1328336

**Published:** 2023-11-10

**Authors:** Timotej Turk Dermastia, Ivano Vascotto, Janja Francé, David Stanković, Patricija Mozetič

**Affiliations:** ^1^Marine Biology Station Piran, National Institute of Biology, Piran, Slovenia; ^2^Jožef Stefan International Postgraduate School, Ljubljana, Slovenia; ^3^Department of Organisms and Ecosystems Research, National Institute of Biology, Ljubljana, Slovenia

**Keywords:** *rbcL*, metabarcoding, monitoring, diatoms, population genetics, *Pseudo-nitzschia*, Adriatic

In the published article, there was an error. In section **2. Methods**, *2.3. Amplification and Illumina MiSeq sequencing*, the forward primer 708F-DEG has been incorrectly pasted as the same as the 18S-V9R primer. The paragraph previously stated:

“Two markers were chosen for metabarcoding, namely the 150 base pair (bp) 18S-V9 region and a ~ 312 bp barcode within the *rbcL* chloroplast gene. The primers used to amplify the 18S gene were the universal eukaryotic 18S-V9F (TTGTACACACCGCCCGTCGC) and 18S-V9R (CCTTCYGCAGGTTCACCTAC; Piredda et al., 2017). Libraries for the *rbcL* barcode were built using the diatom-specific primers 708F-DEG (CCTTCYGCAGGTTCACCTAC) and R3-DEG (CCTTCTAATTTACCWACWACWG), both modified from Vasselon et al. (2017a).”

The corrected sentence appears below:

“Two markers were chosen for metabarcoding, namely the 150 base pair (bp) 18S-V9 region and a ~312 bp barcode within the *rbcL* chloroplast gene. The primers used to amplify the 18S gene were the universal eukaryotic 18S-V9F (TTGTACACACCGCCCGTCGC) and 18S-V9R (CCTTCYGCAGGTTCACCTAC; Piredda et al., 2017). Libraries for the *rbcL* barcode were built using the diatom-specific primers 708F-DEG (AGGTGAAGYWAAAGGTTCWTAYTTAAA) and R3-DEG (CCTTCTAATTTACCWACWACWG), both modified from Vasselon et al. (2017a).”

The authors apologize for this error and state that this does not change the scientific conclusions of the article in any way. The original article has been updated.

