# Supplementary material for: Evaluation of the rbcL marker for metabarcoding of marine diatoms and inference of population structure of selected genera
Source: Front Microbiol. 2023 Mar 2;14:1071379. doi: 10.3389/fmicb.2023.1071379 (PMC10026700; doi:10.3389/fmicb.2023.1071379)

Supplementary Material

1 Supplementary Data

Data availability:

The datasets generated and analysed for this study can be found in Zenodo [https://zenodo.org/record/7064747] and via ENA/GenBank under BioProject Accession PRJEB57391 and individual FASTQ files under accessions PRJEB57360 (18S-V9) and PRJEB57359 (*rbcL*).

Code Piece 1: GenBank searchterm to retrieve 18S and *rbcL* sequences

```
rbcL[Title] AND ((((("Eukaryota"[Organism] NOT predicted[Title] NOT environmental[Title] NOT uncultured[Title]) NOT unclassified[Title]) NOT unverified[Title]) NOT putative[Title]) NOT uncharacterized[Title]) NOT predicted[Title]) NOT scaffold[Title] NOT hypothetical[Title] AND (biomol_genomic[PROP] AND is_nuccore[filter] AND chloroplast[filter])

18S[Title] AND ((((("Bacillariophyta"[Organism] NOT predicted[Title] NOT environmental[Title] NOT uncultured[Title]) NOT unclassified[Title]) NOT unverified[Title]) NOT putative[Title]) NOT uncharacterized[Title]) NOT predicted[Title]) NOT scaffold[Title] NOT hypothetical[Title] AND (biomol_genomic[PROP] AND is_nuccore[filter])
```

2 Supplementary Tables

Supplementary Table 1: Sample summary. QF amplicons are merged forward and reverse reads, denoised with dada2 and with removed chimeras.

Sample ID	Marker	Raw reads	QF merged amplicons	% retained	Diatom amplicons	%	Date	Filter	Depth (m)
561461F532487	18S	40544	35999	88.8	112	0.3	17-Sep-19	0.8 µm	0
561462F532488	18S	43159	37015	85.8	120	0.3	17-Sep-19	0.8 µm	5
723820F710998	*rbcL*	100899	19476	19.3	16037	82.3	17-Sep-19	0.8 µm	0
723821F710999	*rbcL*	105447	21987	20.9	18885	85.9	17-Sep-19	0.8 µm	5
561463F532490	18S	39243	34812	88.7	704	2.0	16-Oct-19	0.8 µm	5
561464F532491	18S	45369	37121	81.8	3222	8.7	21-Nov-19	0.8 µm	0
561465F532492	18S	53264	44330	83.2	2744	6.2	21-Nov-19	0.8 µm	5
723822F711000	*rbcL*	135333	30978	22.9	22368	72.2	21-Nov-19	0.8 µm	0
723823F711001	*rbcL*	141845	34261	24.2	23506	68.6	21-Nov-19	0.8 µm	5
561466F532493	18S	48854	39480	80.8	3988	10.1	16-Dec-19	0.8 µm	0
561467F532494	18S	47104	39294	83.4	3644	9.3	16-Dec-19	0.8 µm	5
723824F711002	*rbcL*	27288	8337	30.6	5881	70.5	16-Dec-19	0.8 µm	0
723825F711003	*rbcL*	183453	37869	20.6	23305	61.5	16-Dec-19	0.8 µm	5
561468F532495	18S	70159	59664	85.0	4371	7.3	15-Jan-20	0.8 µm	0
561469F532496	18S	62480	53729	86.0	3601	6.7	15-Jan-20	0.8 µm	5
723826F711004	*rbcL*	186480	41832	22.4	36060	86.2	15-Jan-20	0.8 µm	0
723827F711005	*rbcL*	181016	37530	20.7	33380	88.9	15-Jan-20	0.8 µm	5
561470F532497	18S	46981	40055	85.3	1556	3.9	2-Feb-20	0.8 µm	0
561471F532498	18S	41452	34871	84.1	1332	3.8	2-Feb-20	0.8 µm	5
723828F711006	*rbcL*	164157	42031	25.6	36571	87.0	2-Feb-20	0.8 µm	0
723829F711007	*rbcL*	143107	40850	28.5	35623	87.2	2-Feb-20	0.8 µm	5

561479F532510	18S	53856	49419	91.8	3806	7.7	13-Oct-20	0.8 µm	Integrated
723830F711008	*rbcL*	188234	38330	20.4	38330	100.0	13-Oct-20	0.8 µm	Integrated

Supplementary Table 2: Species list recovered by rbcL (LULU+BLAST)

**Family**	**Species**	**Status of taxon in Gulf of Trieste (microscopy and barcoding)**
Bacillariaceae	*Cylindrotheca closterium*	✓
Bacillariaceae	*Nitzschia spp.*	✓
Bacillariaceae	*Psammodictyon pustulatum*	✓
Bacillariaceae	*Pseudo-nitzschia cf. delicatissima*	NEW
Bacillariaceae	*Pseudo-nitzschia delicatissima*	✓
Bacillariaceae	*Pseudo-nitzschia galaxiae*	✓
Bacillariaceae	*Pseudo-nitzschia mannii*	✓
Bacillariaceae	*Pseudo-nitzschia subfraudulenta*	✓
Bacillariaceae	*Pseudo-nitzschia fraudulenta*	✓
Bacillariaceae	*Pseudo-nitzschia linea*	NEW
Amphipleuraceae	*Halamphora banzuensis*	*H. calliformis*
Naviculaceae	*Meuniera membranacea*	✓
Naviculaceae	*Navicula sp.*	✓
Pleurosigmataceae	*Pleurosigma sp.*	✓
Suriellaceae	*Petrodictyon gemma*	✓
Suriellaceae	*Surirella sp.*	✓
Chaetocerotacaea	*Bacteriastrum furcatum*	✓

Chaetocerotacaea	*Bacteriastrum jadranum*	✓
Chaetocerotacaea	*Chaetoceros acadianus*	NEW
Chaetocerotacaea	*Chaetoceros brevis*	✓
Chaetocerotacaea	*Chaetoceros cf. tortissimus*	✓
Chaetocerotacaea	*Chaetoceros cf. vixvisibilis*	✓
Chaetocerotacaea	*Chaetoceros costatus*	✓
Chaetocerotacaea	*Chaetoceros curvisetus*	✓
Chaetocerotacaea	*Chaetoceros dayaensis*	NEW
Chaetocerotacaea	*Chaetoceros decipiens*	✓
Chaetocerotacaea	*Chaetoceros diversus*	✓
Chaetocerotacaea	*Chaetoceros eibenii*	✓
Chaetocerotacaea	*Chaetoceros lauderi*	✓
Chaetocerotacaea	*Chaetoceros protuberans*	✓
Chaetocerotacaea	*Chaetoceros rostratus*	✓
Chaetocerotacaea	*Chaetoceros socialis*	✓
Chaetocerotacaea	*Chaetoceros tenuissimus*	✓
Chaetocerotacaea	*Chaetoceros throndsenii*	✓
Chaetocerotacaea	*Chaetoceros tortissimus*	✓
Leptocylindraceae	*Leptocylindrus danicus*	✓
Coscinodiscaceae	*Coscinodiscus sp.*	✓
Heliopeltaceae	*Actinoptychus octonarius*	possibly as *Actinocyclus* genus
Paraliaceae	*Paralia sulcata*	✓
Rhizosoleniaaceae	*Dactyliosolen blavyanus*	✓

Rhizosoleniaaceae	*Guinardia flaccida*	✓
Rhizosoleniaaceae	*Guinardia striata*	✓
Rhizosoleniaaceae	*Pseudosolenia calcar-avis*	✓
Rhizosoleniaaceae	*Rhizosolenia formosa*	✓
Rhizosoleniaaceae	*Rhizosolenia imbricata*	✓
Stephanodiscaceae	*Cyclotella choctawhatcheeana*	only genus
Stephanodiscaceae	*Stephanodiscus hantzschii*	only genus
Lauderiaceae	*Lauderia sp.*	*Lauderia annulata*
Thalassiosiraceae	*Minidiscus trioculatus*	NEW
Thalassiosiraceae	*Thalassiosira angulata*	only genus
Thalassiosiraceae	*Thalassiosira cf. eccentrica*	only genus
Thalassiosiraceae	*Thalassiosira mediterranea*	only genus
Thalassiosiraceae	*Thalassiosira oceanica*	only genus
Thalassiosiraceae	*Thalassiosira profunda*	only genus
Thalassiosiraceae	*Thalassiosira pseudonana*	only genus
Thalassiosiraceae	*Thalassiosira sundarbana*	only genus
Hemiaulaceae	*Thalassionema frauenfeldii*	✓
Hemiaulaceae	*Cerataulina pelagica*	✓
Cymatosiraceae	*Eucampia cornuta*	✓
Cymatosiraceae	*Arcocellulus mammifer*	NEW
Cymatosiraceae	*Extubocellulus spinifer*	NEW
Cymatosiraceae	*Minutocellus polymorphus*	NEW
Cymatosiraceae	*Papiliocellulus elegans*	NEW
Cymatosiraceae	*Papiliocellulus simplex*	NEW

Lithodesmiaceae	*Lithodesmium intricatum*	✓

Supplementary Table 3: List of *Chaetoceros* species identified by microscopy during harmful algae monitoring. + indicates a mild bloom, ++ indicates a strong bloom.

September 2019	October 2019	November 2019	December 2019	January 2020	February 2020	October 2020
C. cf. vixvisibilis	*C. lauderi*		*C. socialis*	*C. curvisetus* ++	*C. pseudocurvisetus*	*C. decipiens*
C. anastomosans	*C. brevis*			*C. danicus* +		*C. socialis*
C. socialis	*C. dydimus*			*C. affinis*		*C. vixvisibilis*
C. lauderi	*C. curvisetus*			*C. dydimus*		*C. curvisetus*
C. lorenizanum	*C. decipiens*			*C. vixvisibilis*		*C. rostratus*
	C. socialis					*C. lauderi*
	C. tortissimus					*C. dydimus*
						C. affinis
						C. similis
						C. tortissimus
						C. danicus

Figure S1: Diatom species accumulation curves from 18S-V9 (NBC50 taxonomy) and *rbcL* (LULU curated, BLAST taxonomy)

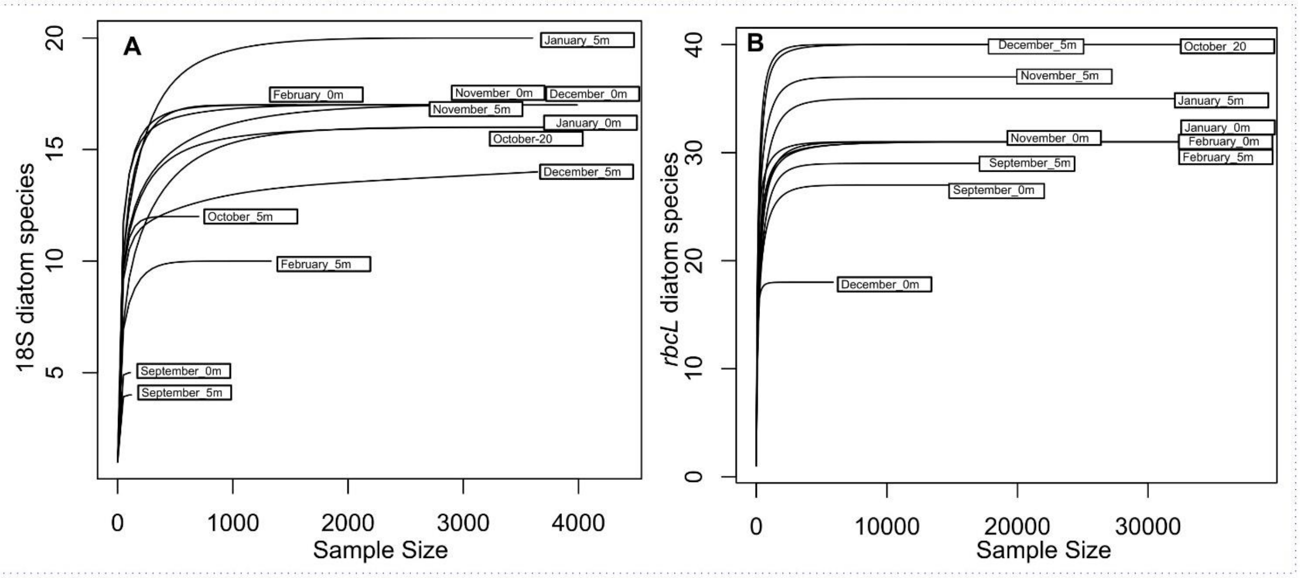

Figure S2: Significance test of different diversity indexes for each combination. Red color indicates significant differences (p<0.05) while blue indicates non-significant differences as determined by the TukeyHSD tests.

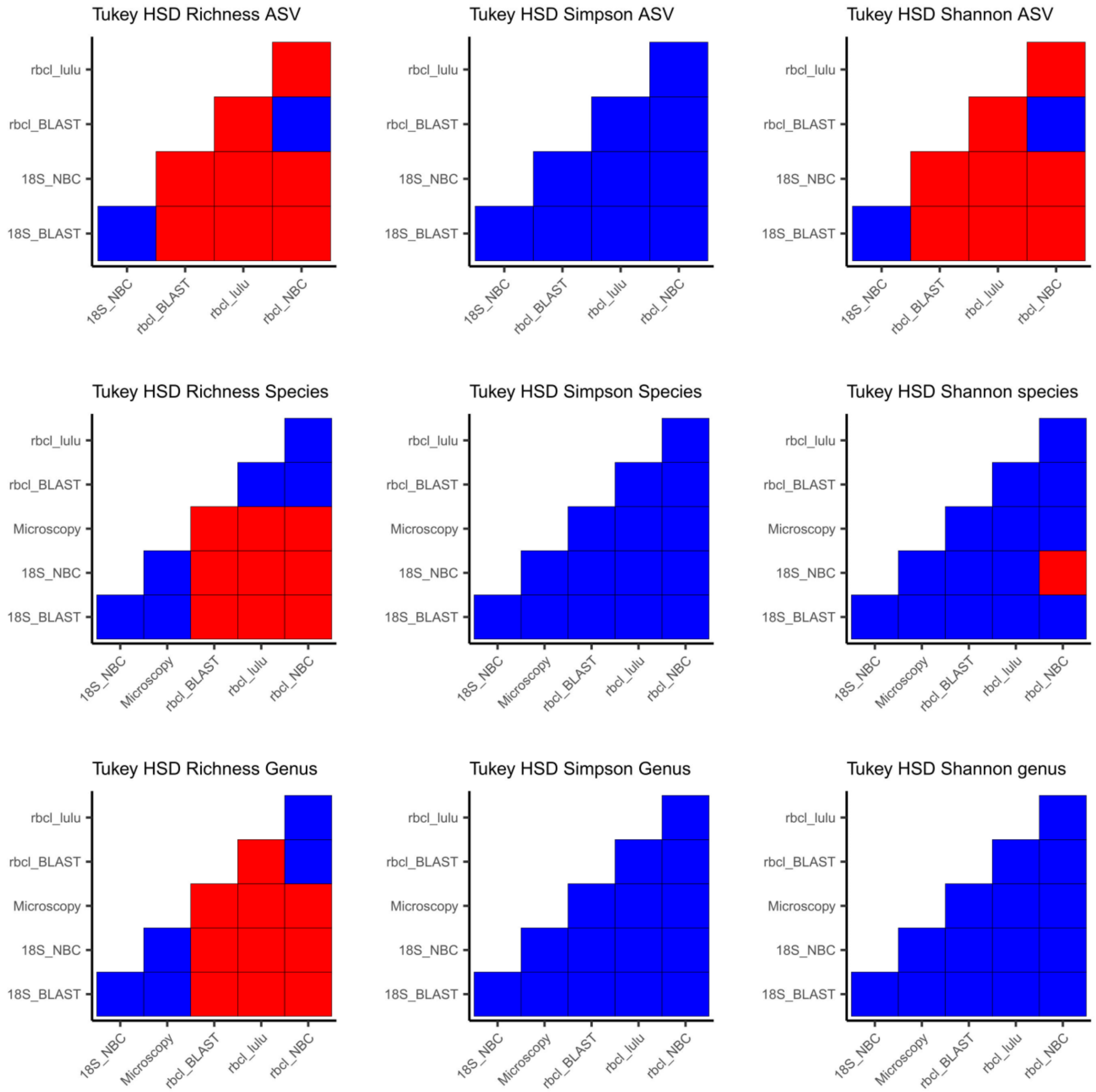

Figure S3: Unicellular protist assemblage in the studied months, obtained with 18S-V9. Diatoms represent the majority of reads classified as Ochrophyta.

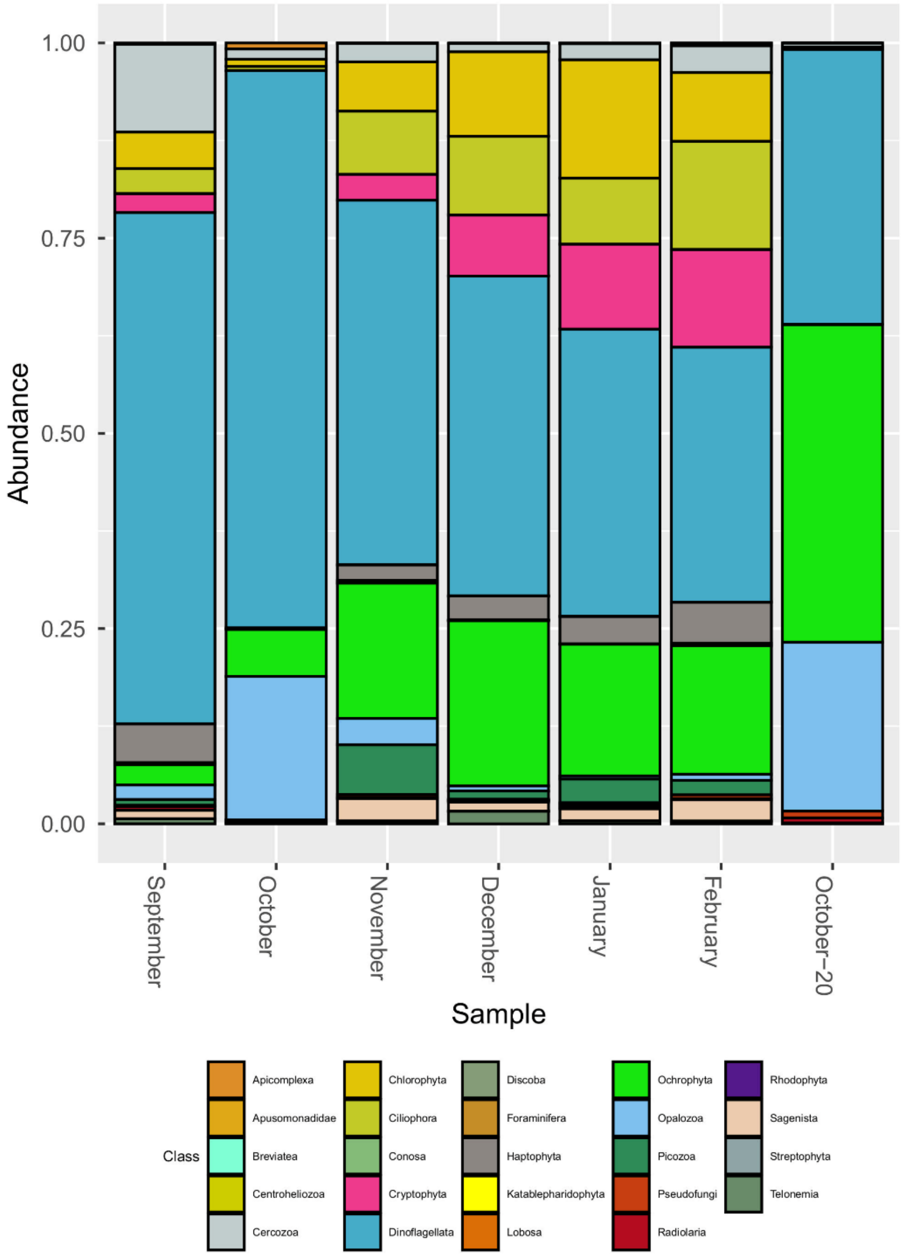

Figure S4: PCA of CLR transformed values and detrended correspondence analysis including microscopy samples.

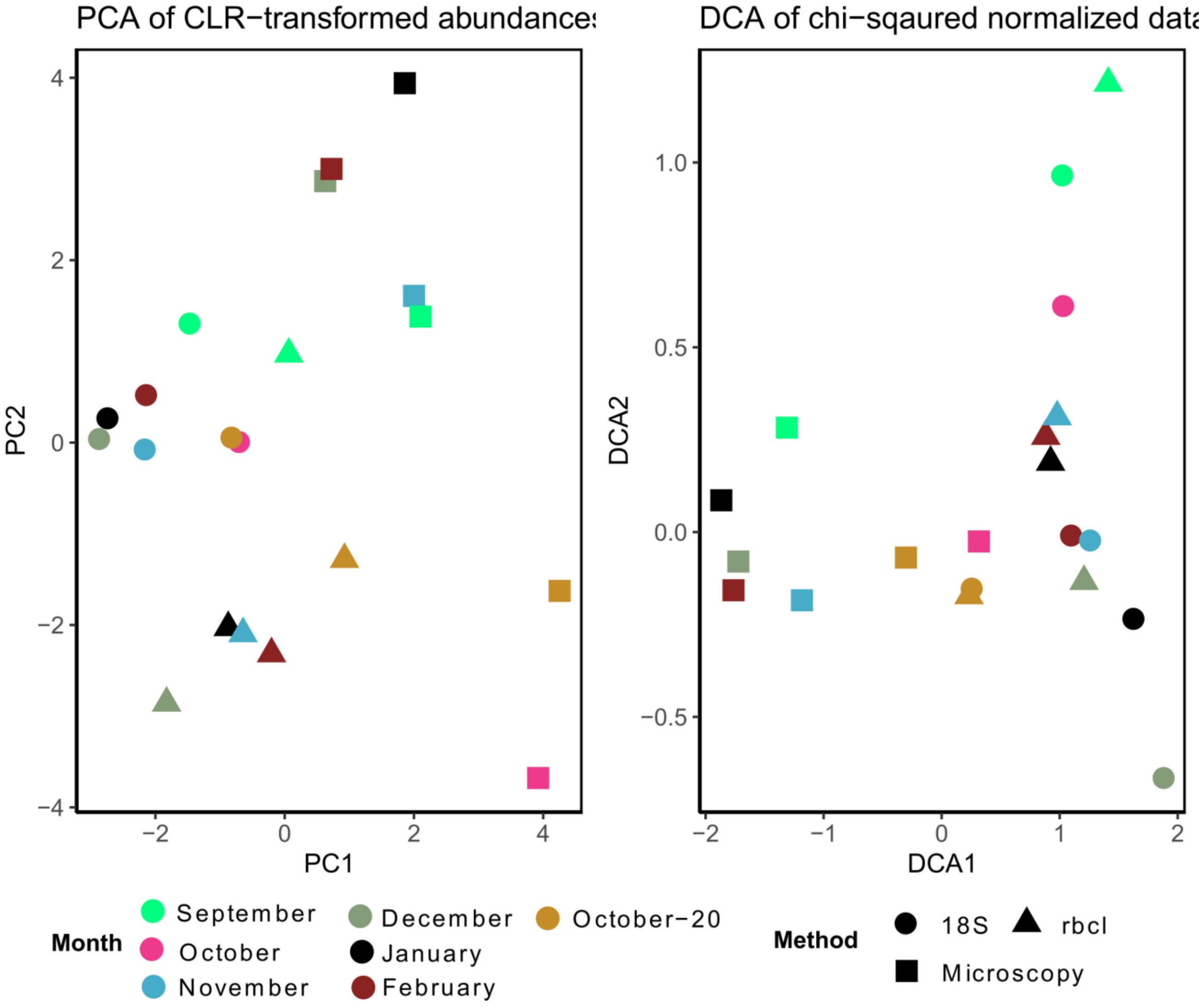

Supplement: Supplementary file 1 [file Data_Sheet_1.pdf]
